# Intrinsic Noise Profoundly Alters the Dynamics and Steady State of Morphogen-Controlled Bistable Genetic Switches

**DOI:** 10.1371/journal.pcbi.1005154

**Published:** 2016-10-21

**Authors:** Ruben Perez-Carrasco, Pilar Guerrero, James Briscoe, Karen M. Page

**Affiliations:** 1 Department of Mathematics, University College London, Gower Street, London WC1E 6BT, UK; 2 The Francis Crick Institute, 1 Midland Road, London, NW1 1AT, UK; Utrecht University, THE NETHERLANDS

## Abstract

During tissue development, patterns of gene expression determine the spatial arrangement of cell types. In many cases, gradients of secreted signalling molecules—morphogens—guide this process by controlling downstream transcriptional networks. A mechanism commonly used in these networks to convert the continuous information provided by the gradient into discrete transitions between adjacent cell types is the genetic toggle switch, composed of cross-repressing transcriptional determinants. Previous analyses have emphasised the steady state output of these mechanisms. Here, we explore the dynamics of the toggle switch and use exact numerical simulations of the kinetic reactions, the corresponding Chemical Langevin Equation, and Minimum Action Path theory to establish a framework for studying the effect of gene expression noise on patterning time and boundary position. This provides insight into the time scale, gene expression trajectories and directionality of stochastic switching events between cell states. Taking gene expression noise into account predicts that the final boundary position of a morphogen-induced toggle switch, although robust to changes in the details of the noise, is distinct from that of the deterministic system. Moreover, the dramatic increase in patterning time close to the boundary predicted from the deterministic case is substantially reduced. The resulting stochastic switching introduces differences in patterning time along the morphogen gradient that result in a patterning wave propagating away from the morphogen source with a velocity determined by the intrinsic noise. The wave sharpens and slows as it advances and may never reach steady state in a biologically relevant time. This could explain experimentally observed dynamics of pattern formation. Together the analysis reveals the importance of dynamical transients for understanding morphogen-driven transcriptional networks and indicates that gene expression noise can qualitatively alter developmental patterning.

## Author Summary

The bistable switch, a common regulatory sub-network, is found in many biological processes. It consists of cross-repressing components that generate a switch-like transition between two possible states. In developing tissues, bistable switches, created by cross-repressing transcriptional determinants, are often controlled by gradients of secreted signalling molecules—morphogens. These provide a mechanism to convert a morphogen gradient into stripes of gene expression that determine the arrangement of distinct cell types. Here we use mathematical models to analyse the temporal response of such a system. We find that the behaviour is highly dependent on the intrinsic fluctuations that result from the stochastic nature of gene expression. This noise has a marked effect on both patterning time and the location of the stripe boundary. One of the techniques we use, Minimum Action Path theory, identifies key features of the switch without computationally expensive calculations. The results reveal a noise driven switching wave that propels the stripe boundary away from the morphogen source to eventually settle, at steady state, further from the morphogen source than in the deterministic description. Together the analysis highlights the importance dynamics in patterning and demonstrates a set of mathematical tools for studying this problem.

## Introduction

Tissue development relies on the spatially and temporally organised allocation of cell identity, with each cell adopting an identity appropriate for its position within the tissue. In many cases, cellular decisions are made by transcriptional networks controlled by extrinsic signals [1–3]. These signals, usually termed morphogens, spread from a localised source within, or adjacent to, the developing tissue to form a spatial gradient that becomes the patterning axis of the tissue. Cells are sensitive to the level of the morphogen and respond by producing a set of discrete gene expression stripes at different distances from the morphogen source [1, 4].

A transcriptional mechanism capable of the analogue to digital conversion necessary to transform the continuous morphogen gradient into distinct domains of gene expression is the so-called *genetic toggle switch* [5, 6]. This sub-network, present in many biological contexts, consists of cross-repression between sets of transcriptional determinants that are expressed mutually exclusively in alternative cell identities [7–11]. Thus the expression of one set of factors represses the alternative identity and vice versa, creating a bistable switch [12–14]. This mechanism has been extensively studied as a way for cells to make decisions and produce distinct outputs in response to biological signals [15–18]. In the case of tissue patterning, a morphogen gradient can modulate the production rates of one or more genes that comprise the switch. This controls the position along the patterning axis at which the switch creates a boundary between cell identities [5, 11]. Moreover, the principle can be extended to incorporate multiple morphogen controlled toggle switches, each producing a boundary at a distinct position, hence explaining the multiple stripes of gene expression generated in a tissue [19, 20]

Mathematical models of morphogen-controlled toggle switches that reproduce the ultrasensitivity necessary to create discrete gene expression boundaries also generate a temporal sequence of gene expression, prior to reaching steady state, that recapitulates the final spatial pattern [11, 18, 20]. This sequence and its timing is a consequence of the inherent dynamical properties of the bistable switch [17, 20, 21]. Strikingly, the temporal behaviour predicted by the models corresponds to experimental observations of gene expression timing in several developing tissues [11, 22–25] and has led to the suggestion that it explains temporal features of morphogen-controlled tissue patterning [20].

Despite the apparent agreement between mathematical models and experimental observations, whether the models correctly identify the biological mechanism responsible for the dynamics and boundary positioning in morphogen-patterned tissues remains unclear. In particular, most current models (excepting [25]) are deterministic and have not addressed whether stochastic fluctuations that arise from noisy gene expression qualitatively alter the behaviour of morphogen-controlled toggle switches. Fluctuations in the production and degradation rates of mRNA and protein molecules in individual cells can lead to substantial molecular heterogeneity [26–29]. Moreover, genes switch between active and inactive states resulting in bursts of transcription interspersed by refractory periods in which transcription is suppressed [26, 30–32]. The intrinsic variations introduced by these processes could facilitate spontaneous transitions between different cell states, see for example [16, 33–39]. The effect of stochastic fluctuations on the position and precision of boundaries needs to be explored as previous work has suggested, counter-intuitively, that noise can sharpen boundaries in a tissue [1, 25]. Thus in addition to the effect of gene expression noise on the steady state of a genetic toggle switch, understanding how stochastic fluctuations influence the temporal behaviour of switching along a morphogen patterning axis is necessary.

Here we develop a theoretical framework to investigate the dynamics of morphogen-controlled genetic toggle switches and analyse how noise in gene expression affects tissue patterning by this mechanism. We show that exact numerical simulations of the kinetic reactions, the corresponding Chemical Langevin Equation, and Minimum Action Path theory provide insight into the trajectory, dynamics and directionality of stochastic switching between states of a bistable switch. First, we introduce the deterministic and stochastic models of the morphogen-controlled toggle switch. We show how patterning time varies with the morphogen signal in the deterministic switch and how intrinsic fluctuations alter the patterning time in the monostable zone. We continue by demonstrating how stochastic switching positions the gene expression boundary within the bistable zone, exploring how the typical number of proteins and expression bursts shape the stochastic switching process. Next we show how this analysis suggests that the expression of the morphogen-induced gene is activated as a wave that travels away from the morphogen source, setting the position of the pattern boundary. How the velocity and sharpness of the wavefront depends on the parameters of the system is analysed in the last section of the results. Finally, we discuss these results in the context of tissue patterning, as well as the broader utility of our theoretical framework.

## Models

**Fig 1:**
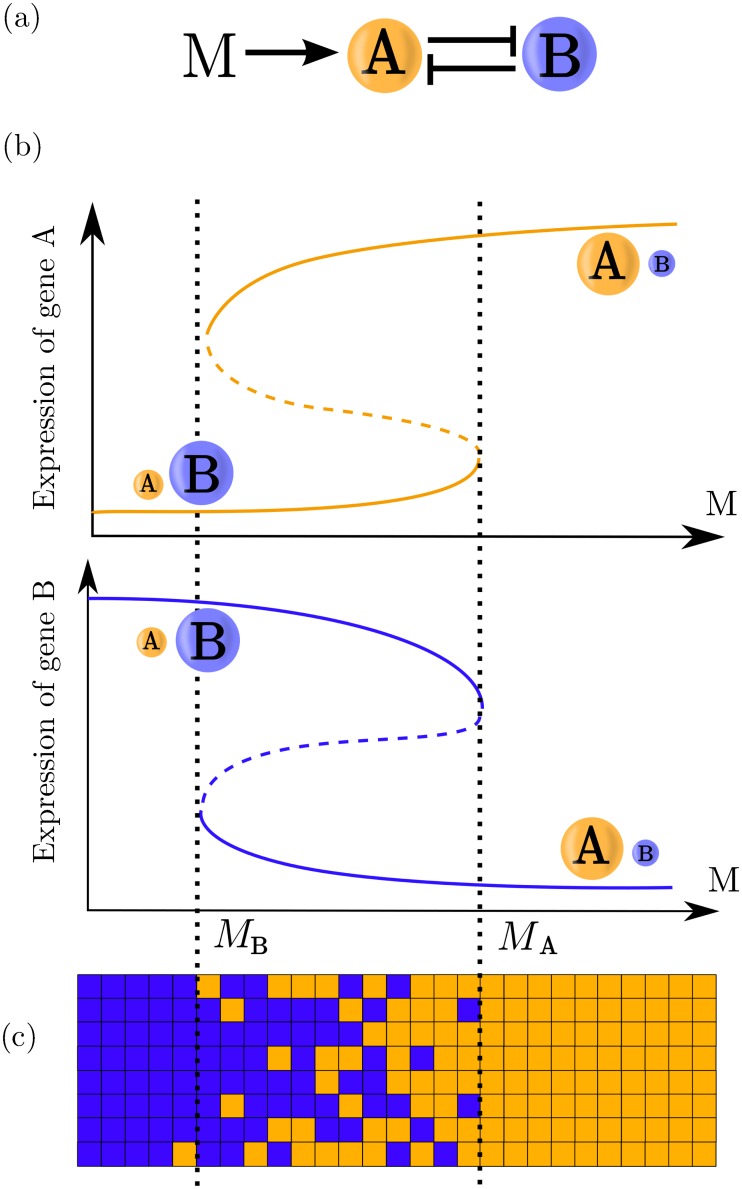
Bistable switch patterning schematic. (a) Genetic network showing the cross-repression of genes *A* and *B*. The signal *M* activates gene *A* changing the available steady states. (b) Stability diagrams for expression of genes A and B showing the available steady states for different values of the signal. Stable steady states are indicated with solid lines, while the unstable steady state (in this case a saddle point) is indicated with a dashed line. (c) A schematic of tissue patterning with the genetic toggle switch: In the monostable zones (to the left and right of the vertical dotted lines) cells will adopt the state B or A, respectively. The state adopted by a cell in the bistable zone (between the vertical dotted lines) will be determined by the history of the signal and by stochastic effects.

In order to characterise the dynamics of a bistable switch we consider a model in which two genes *A* and *B* repress each other, and a morphogen signal *M* acts as an activator of gene *A* promoting its expression (Fig 1). This is motivated by the developmental biology scenario of a tissue initially expressing a gene *B*, sometimes referred to as the “prepattern”, that is exposed to a graded morphogen *M* which induces expression of the “target” gene *A*. Examples can be found in the vertebrate neural tube, in which cells initially express the transcription factors Irx3 and Pax6. These form cross-repressive interactions with transcription factors Olig2 and Nkx2.2, which are controlled by the Shh morphogen [11]. Similarly in the Drosophila blastoderm, the morphogen Bicoid induces the transcription factors Giant and Slp1 which cross-repress Kruppel and Runt, respectively [40]. In the nomenclature we adopt, the two possible cellular steady state outcomes of the patterning, A and B, are characterised by high levels of expression of one gene and lower levels of the other. For intermediate morphogen signalling levels, both states are stable, with the region of bistability given by *M*_*B*_ < *M* < *M*_*A*_. In this region, the cellular outcome will depend on the initial conditions and the history of the morphogen signal. In response to the graded distribution of the morphogen *M*, this model is capable of generating two abutting stripes of gene expression. This mechanism can be readily extended to accommodate additional stripes at different morphogen levels [11, 19, 20].

Even in the simple scenario, however, there are many possible mechanisms regulating two-gene interactions, these can act at transcription, translation or post-translational levels. Despite this, the essential mechanism of patterning reduces to the same bistable switch sub-network (Fig 1) [11, 12, 14, 20, 25, 41].

In the current study we consider that protein production occurs on a much slower time scale than transcription factors binding and unbinding to the enhancer, RNA polymerase binding and unbinding the promoter, transcription and mRNA degradation. Under this assumption the production of each protein can be considered proportional to the probability of finding the polymerase bound to its promoter ${\tilde{p}}_{i}$ with *i* ∈ *A*, *B*. The rate of change in time of protein concentration *n*_*i*_(*t*) can then be described as

$$\begin{array}{ccc} {\dot{n}}_{A} & = & \alpha_{A}{\tilde{p}}_{A}\left(M,n_{B}\right)-\delta_{A}n_{A}\\ {\dot{n}}_{B} & = & \alpha_{B}{\tilde{p}}_{B}\left(n_{A}\right)-\delta_{B}n_{B}, \end{array}$$ (1)

where *α*_*i*_ is the protein production rate when RNAp is bound to gene *i*, and *δ*_*i*_ is the effective degradation rate of protein *i*. Additionally, the polymerase binding probability ${\tilde{p}}_{A}$ depends on the morphogen signal *M*, since the morphogen controls the production rate of gene *A*. In Eq (1) the concentration of protein is given in arbitrary units that can be related to the actual number of proteins *N*_*i*_ through a multiplicative constant $\tilde{\Omega }$, *i.e.* $N_{i}=n_{i}\tilde{\Omega }$.

Eq (1) can be non-dimensionalised by expressing time in units of $\delta_{A}^{-1}$ and the concentration of proteins in units of *α*_*A*_/*δ*_*A*_ obtaining,

$$\begin{array}{ccc} {\dot{x}}_{A} & = & p_{A}\left(M,x_{B}\right)-x_{A}\\ {\dot{x}}_{B} & = & \alpha p_{B}\left(x_{A}\right)-\delta x_{B}, \end{array}$$ (2)

describing the evolution of the non-dimensional protein expression *x*_*i*_ ≡ *δ*_*A*_*n*_*i*_/*α*_*A*_ where the magnitudes *α* ≡ *α*_*B*_/*α*_*A*_ and *δ* ≡ *δ*_*B*_/*δ*_*A*_ are respectively the relative rates of production and degradation of genes *A* and *B* and the time derivatives are taken with respect to the non-dimensional time. Similarly, the non-dimensional binding probabilities are defined as $p_{A}\left(M,x_{B}\right)\equiv {\tilde{p}}_{A}\left(M,n_{B}\right)$ and $p_{B}\left(x_{A}\right)\equiv {\tilde{p}}_{B}\left(n_{A}\right)$. This non-dimensionalization simplifies the study of the system and also reveals intrinsic properties of the network. For given functions *p*_*i*_, the whole dynamical system behaviour only depends on the parameter ratios *α* and *δ* regardless of the actual values of *α*_*i*_ or *δ*_*i*_. Additionally, the non-dimensionless expression levels of each stable state (${\dot{x}}_{i}\left(x_{i}^{st}\right)=0$) only depend on the ratio *α*/*δ* independent of the actual values of *α* and *δ*,

$$\begin{array}{c} x_{A}^{st}=p_{A}\left(M,x_{B}^{st}\right)\\ x_{B}^{st}=\frac{\alpha }{\delta }p_{B}\left(x_{A}^{st}\right). \end{array}$$ (3)

**Fig 2:**
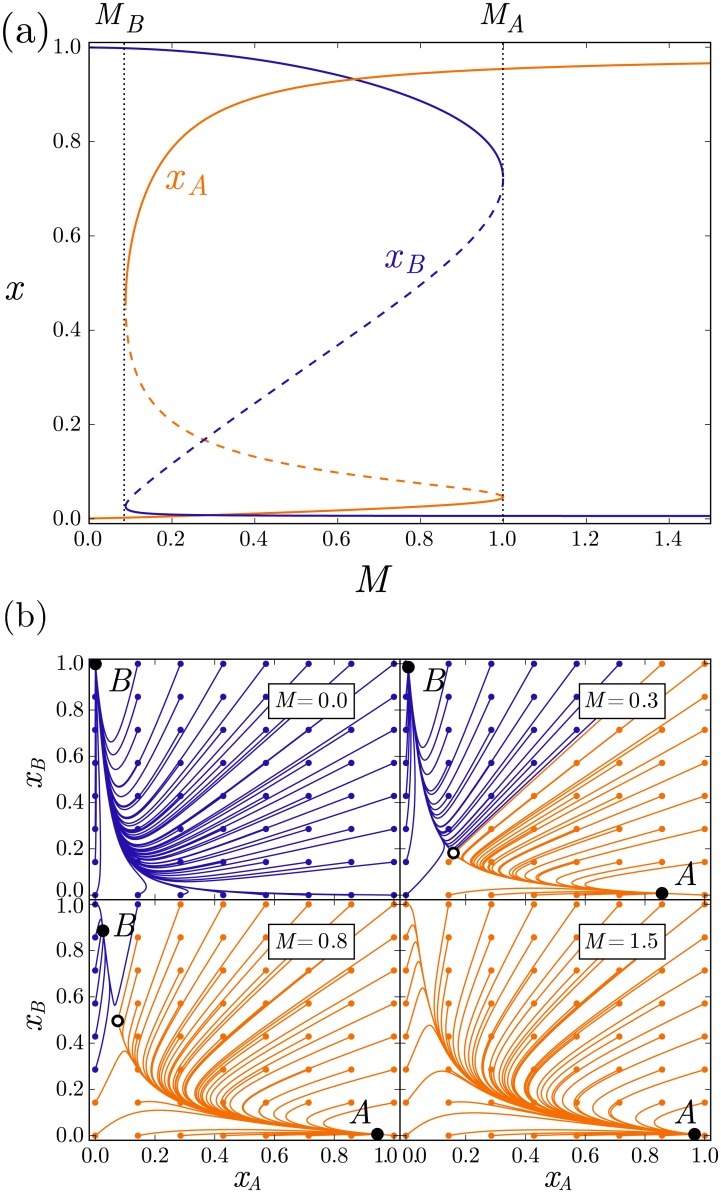
Dynamical behaviour of the bistable switch. (a) Bifurcation diagram of the bistable switch, showing solutions of Eq (3) as a function of the signal *M* for proteins *A* (orange) and *B* (blue). The stable steady states (solid lines) and the saddle points (dashed lines) are indicated. (b) Trajectories for the indicated levels of signal *M*, starting at different initial conditions, marked by coloured circles. The colour indicates the final stationary state converging to state A (orange) or B (blue). Additionally, the stable steady states (black circles), and the saddle point (white circle) are indicated. Parameters used are *α* = *δ* = *ρ*_*A*_ = 1, *ρ*_*B*_ = 1.75 ⋅ 10^−4^, *K*_*A*_ = 10^−3^, *K*_*B*_ = 3 ⋅ 10^−2^, *K*_*M*_ = 1, *f* = 10.0.

The RNAp binding probabilities *p*_*i*_ can be described using statistical physics principles by computing the fraction of possible equilibrium configuration states in which RNAp is bound to the target gene promoter [42]. We will consider the case in which each enhancer has two non-overlapping binding sites for the repressive transcription factor that interact independently and, when occupied, forbid the binding of RNAp [43]. Assuming that the signal reduces the recruiting binding energy of RNAp to the promoter of gene A, and that its effector also has two independent binding sites, also independent of the repressor sites, the probabilities of transcription can be written as,

$$\begin{array}{ccc} p_{A}\left(M,x_{B}\right) & = & {\left(1+\rho_{A}{\left(\frac{1+M/K_{M}}{1+fM/K_{M}}\right)}^{2}{\left(1+\frac{x_{B}}{K_{B}}\right)}^{2}\right)}^{-1}\\ p_{B}\left(x_{A}\right) & = & {\left(1+\rho_{B}{\left(1+\frac{x_{A}}{K_{A}}\right)}^{2}\right)}^{-1}, \end{array}$$ (4)

where *ρ*_*i*_ sets the basal gene activation, *f* controls the activation strength of the signal, *K*_*i*_ is the rescaled equilibrium dissociation constant of protein *i* for its binding site in the relevant enhancer, and *K*_*M*_ the equilibrium dissociation constant of the morphogen effector with the enhancer of gene *A*. All parameters of Eq (4) are positive and *f* > 1. The regulatory Eq (4) returns the usual polynomial ratios that give rise to a sigmoidal response for the activator and the repressor [17, 43, 44] (Fig 2).

The morphogen gradient signal *M* is typically a monotonically decreasing function of the distance from its source. Thus, in contrast to many studies of toggle switches, which focus on the behaviour for some fixed values of the signal, the patterning problem requires us to understand how the response varies along a continuous gradient of a signal. Without any loss of generality, the precise spatial dependence will be omitted and we just consider the outcome in response to a continuous signal *M*. Some previous studies introduce a more complex range of interaction between morphogen gradients and cells, either by considering spatial coupling between cells [14, 25] or by direct interpretation of the linear spatio-temporal changes of the morphogen gradient [45]. In contrast, our choice to remove cell-cell interaction or signalling dynamics aims to reveal fundamental patterning properties of a bistable switch ubiquitous in similar multistable regulatory systems.

### Stochastic dynamics

Protein production and degradation described in Eq (1) give a deterministic description of the bistable switch dynamics. This deterministic description is the coarse grained outcome of the underlying single production and degradation stochastic events that generate intrinsic noise in the network [26, 27]. This random component can be included in the expression dynamics by the Chemical Langevin Equation (CLE) approximation, that introduces the intrinsic fluctuations as the addition of a multiplicative noise term to the deterministic description [46],

$$\begin{array}{ccc} {\dot{x}}_{A} & = & p_{A}-x_{A}+\sqrt{\nu_{A}p_{A}+x_{A}}\;\xi_{A}\left(t\right)\\ {\dot{x}}_{B} & = & \alpha p_{B}-\delta x_{B}+\sqrt{\nu_{B}\alpha p_{B}+\delta x_{B}}\;\xi_{B}\left(t\right) \end{array}$$ (5)

where *ξ*_*i*_(*t*) is a Gaussian white noise with zero average and is delta-correlated:

$$\begin{array}{ccc} \left\langle \xi_{i}\left(t\right)\right\rangle & = & 0,\\ \left\langle \xi_{i}\left(t\right)\xi_{j}\left(t^{\prime }\right)\right\rangle & = & \delta_{A}\alpha_{A}^{-1}{\tilde{\Omega }}^{-1}\delta \left(t-t^{\prime }\right)\delta_{ij}\\ & \equiv & \Omega^{-1}\delta \left(t-t^{\prime }\right)\delta_{ij}. \end{array}$$ (6)

Here *δ*_*ij*_ is Kronecker’s delta, *δ*(*t* − *t*′) is Dirac’s delta, and Ω is the volume parameter relating concentrations with number of molecules ($N_{A}=n_{A}\tilde{\Omega }=x_{A}\Omega$), which also allows us to express the rates in terms of absolute changes in protein number per unit of time for the different reaction channels. The deterministic result Eq (2) is recovered in the limit Ω → ∞ [27, 46]. On the other hand, the parameters *ν*_*i*_ introduce the stoichiometries of the production reactions, as a first approximation to account for the effects of expression bursts [46] (for more details see S1 Text).

The introduction of parameters *ν*_*i*_ and Ω does not perturb the deterministic landscape, which only depends on *p*_*i*_, *α* and *δ*. Different *ν*_*i*_ and Ω will result in different noise dependence with the regulatory functions and provide a natural mechanism to change fate determination while keeping the same macroscopic description Eq (1). The sources of noise described reproduce the effects of intrinsic fluctuations arising from production and degradation events of the proteins, through the stochastic parameters Ω, *ν*_*A*_ and *ν*_*B*_. Other sources of noises, such as noise in the morphogen signal, may be of relevance in specific biological scenarios and will require specific formulations of kinetic equations and CLE dynamics.

## Results

### The patterning time of a deterministic bistable switch varies with morphogen level

**Fig 3:**
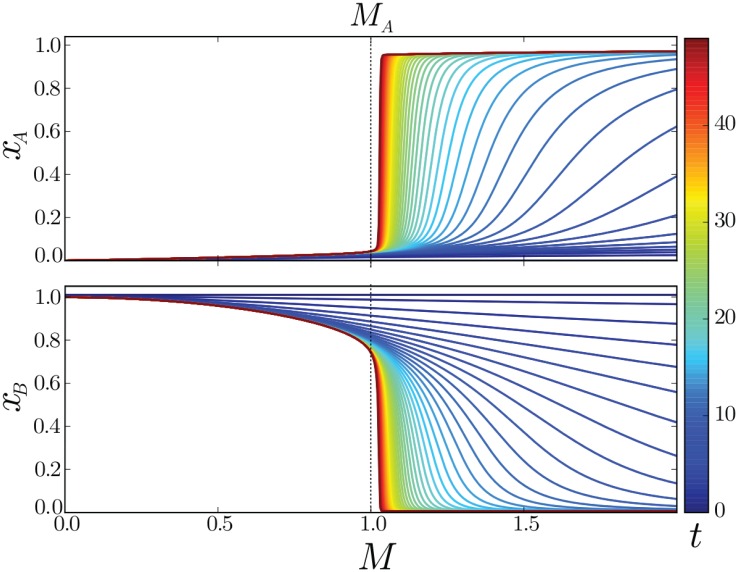
Dynamics of spatial pattern formation in the deterministic case. Each line shows the levels of transcription factors A (top) and B (bottom) as a function of morphogen signal (*M*) at the times indicated by the heatmap (right). The change in gene expression becomes more step-like over time. The closer the signal is to the bifurcation level *M* = *M*_*A*_ = 1, the slower is the rate of change in transcription factor levels; this is a signature of the “dynamical ghost”. Initial conditions are *x*_*A*_ = 0 and *x*_*B*_ = 1.0, the rest of the parameters are those of Fig 2.

For a genetic toggle switch such as that described by Eq (1), bistability means that the final steady state is determined not only by the level of the signal but also the initial condition of the system. In the absence of noise, the response of the system to the signal *M* can be divided into a bistable regime (*M*_*B*_ < *M* < *M*_*A*_) where the steady state is dependent on the initial conditions, and two monostable regimes (*M* > *M*_*A*_ and *M* < *M*_*B*_) where the final state of the system, *A* or *B*, is independent of the initial conditions. The way in which the system switches steady state is by application of a level of signal outside of the bistability zone, *M* > *M*_*A*_ for an initial state B, and *M* < *M*_*B*_ for initial state A (Fig 2). To conform to the tissue patterning paradigm, we assume that prior to the application of the morphogen signal, the “prepattern” gene, *B*, is active homogeneously throughout the tissue. The establishment of the morphogen gradient results in the activation of the “target” gene *A* at positions at which the morphogen concentration exceeds the threshold (*M* > *M*_*A*_) [11, 40] (Fig 3).

The deterministic description of the patterning process is thus reduced to understanding the evolution towards the stable state A in zones of the tissue where *M* > *M*_*A*_. For these positions, state A is not reached immediately but will undergo a transient expression described by Eq (2). For convenience we refer to the time it takes for a cell at a certain signal *M* to change its expression state as the patterning time, *T*. It is important to note that this is not meant to imply that developmental patterning of the tissue requires such times to be reached or that the biological pattern requires all the cells to reach their steady state expression. Instead, it indicates the time during which the pattern of gene expression is changing along tissue. The higher the morphogen concentration the faster gene expression changes, saturating at a minimal characteristic patterning time *T*_*c*_. In contrast, the switch to state *A* becomes slower the closer the signal is to the threshold signal *M*_*A*_, where levels of gene *A* remain low for a long time before it is expressed (*T* ≫ *T*_*c*_) (Fig 3 and S1 Fig). This marked increase in the patterning time is a signature of the mechanism by which the stability of state B is lost at *M* = *M*_*A*_ (saddle-node bifurcation); around *M*_*A*_ the eigenvalues determining stability of *B* vary smoothly with *M* and hence close to *x*^*st*^(*M*_*A*_) the dynamics are very slow (S2 Fig) [20]. This feature has been termed a “dynamical ghost” in recognition that a vestige of the steady state is present in the dynamics of the system near the bifurcation point [21]. This property is a signature of the positive feedback loop present in the bistable switch and is not present in a mechanism that relies solely on strongly cooperative activation of *A* by *M* (See S1 Text).

### Intrinsic fluctuations accelerate cell fate change in the monostable zone

The inherent fluctuations in the biochemical events that control gene expression, such as production and degradation events, introduce stochasticity into the expression dynamics (*e.g.* [28, 29]) (S1 Text). Such fluctuations introduce variability not only in the transient genetic expression (S1 Fig), but also in the average patterning times.

**Fig 4:**
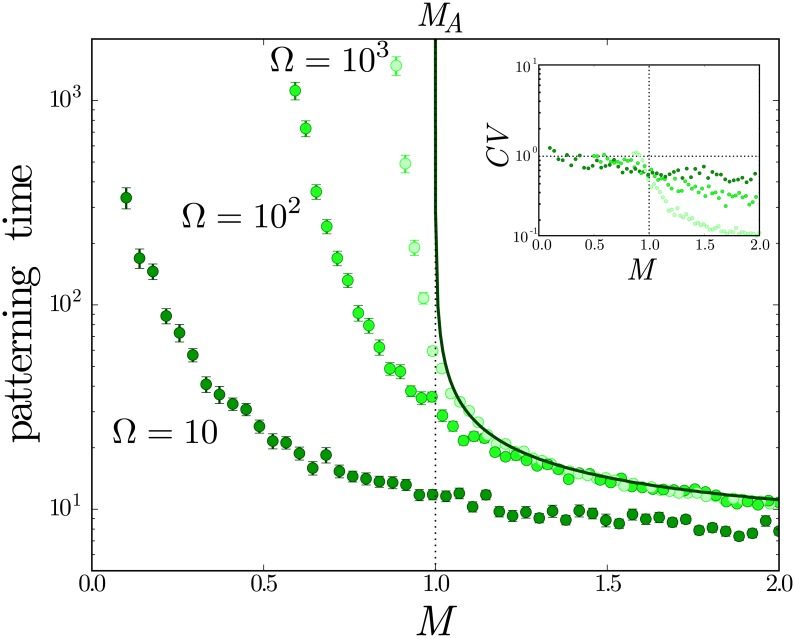
Intrinsic noise changes average patterning time along the tissue. Patterning time, measured as the time to observe a response *x*_*A*_ > 0.9, for the deterministic case (solid line) indicates that the time it takes for a response increases substantially as *M* approaches the deterministic steady state boundary at *M* = *M*_*A*_ = 1. The patterning times of stochastic simulations with different values of Ω (circles) are shown and illustrate that change of cellular expression state can occur within the bistable region, 0.1 < *M* < 1.0. Smaller values of Ω (higher levels of noise) result in decreased patterning times. Each point corresponds to the average mean first passage time of 100 CLE realisations with initial conditions *x*_*A*_ = 0 and *x*_*B*_ = 1.0. Error bars correspond to standard error of the mean. Stochastic parameters are *ν*_*A*_ = *ν*_*B*_ = 1 and Ω = 100, the rest of parameters are those of Fig 2. Inset) Coefficient of variation of the patterning time.

We first focused on how patterning time is altered in the monostable zone where *M* > *M*_*A*_. Comparison with the deterministic simulations indicate that the average patterning times for large values of the signal (*M* ≫ *M*_*A*_) are not affected by intrinsic fluctuations. By contrast, however, for values close to *M*_*A*_, the patterning time is markedly lower in the stochastic simulations compared to the deterministic model (Fig 4). Close to the threshold *M*_*A*_, the slow dynamics introduced by the saddle-node bifurcation do not delay the expression dynamics because the intrinsic noise allows the system to explore the dynamical landscape and escape the dynamical ghost. This accelerates the expression change towards the final state while keeping transient expression levels similar to the deterministic ones. Thus stochasticity in gene expression provides a mechanism that counterbalances the slow dynamics associated with the saddle-node bifurcation present in the deterministic system.

We next examined the effects of stochastic fluctuations in the bistable zone. Close to the bifurcation, the stable steady state B is marginally stable and intrinsic noise can result in sufficient repression of gene *B* and activation of gene *A* to switch to the stable steady state A. This stochastic switching occurs via a similar trajectory in gene expression space and on the same time scale as the patterning time in the monostable zone, resulting in an average patterning time that varies continuously between the monostable and bistable zones (Fig 4). Consequently the resulting pattern boundary is no longer located at *M* ≃ *M*_*A*_ but shifts inside the bistable zone. This behaviour is inherent to the bistable switch and is not found in the non-feedback circuit where noise cannot change the position of the pattern boundary, which is always located at *M* ≃ *M*_*A*_ (See S1 Text).

### Stochastic switching positions the pattern boundary inside the bistable zone

Throughout the bistable region of the system, sufficiently large fluctuations will result in spontaneous switching. Such a transition is always possible, with a patterning time that increases super-exponentially as the signal level *M* decreases (Fig 4). Such a marked increase in patterning time can lead to biologically unfeasible switching times for a range of signal levels. In this range, the system will be resilient to intrinsic noise. By contrast, when the time scale of noise-driven switching is comparable to the patterning time of the tissue, the position and precision of the pattern boundary will be altered. Moreover, since the change in patterning time occurs continuously across the bistability threshold *M*_*A*_, stochastic transitions will always be relevant during tissue patterning.

For time scales larger than the degradation rates, the protein expression for each steady state will follow a certain probability distribution around the deterministic steady state. The larger the typical number of molecules defining each steady state (*N*_*i*_ = *x*_*i*_ Ω), the smaller the effect of fluctuations, the narrower the deviations from the deterministic phenotype, and the longer the typical switching times (Fig 4 and S3 Fig). The tails of this distribution will determine the stochastic switchings. In this context, large deviation theory predicts an exponential dependence of the average stochastic switching patterning time on Ω [47, 49]

$$T_{s}=Ce^{\Omega S},$$ (7)

where *C* is a prefactor to the exponential behaviour and S is the action of the stochastic transition. Eq (7) can be related to the Arrhenius law where Ω^−1^ plays the role of temperature, controlling the fluctuations of the expression state, and S plays the role of the activation energy of the transition between different states. Consistent with this, S changes with the level of the morphogen signal and provides a correlate of the dynamical landscape.

In one-dimensional cases, such as the auto-activating bistable switch, the dependence on the signal of the stochastic switching time can be obtained directly from knowledge of the probability distribution in a closed integral form [38, 50, 51]. In multidimensional cases, such as the one we are studying, there is more than one path across the dynamical landscape linking the steady states and finding the values of S requires us to consider the contribution of the different paths. Each path *φ*_*τ*_ of duration *τ* has a different probability that can be written as [47, 49]

$$P\left(\varphi_{\tau }\right)∼e^{-\Omega S(\varphi_{\tau })},$$ (8)

where the exponential dependence on Ω predicts that for large enough numbers of proteins, the stochastic switching process will occur following the neighbourhood of the path φ* that minimises the action,

$$S\equiv S\left(\varphi^{*}\right)=\min_{\tau ,\varphi_{\tau }}S\left(\varphi_{\tau }\right).$$ (9)

Applying the Eikonal exponential dependence of Eq (8) to the CLE describing the stochastic dynamics of the bistable switch (Eq (5)), a closed expression for the action can be obtained describing the stochastic switching process that for a general CLE of the form $\dot{x}=f\left(x\right)+g\left(x\right)\xi \left(t\right)$ gives [47–49],

$$S\left(\varphi_{\tau }\right)=\frac{1}{2}\int_{0}^{\tau }{\left\|{\dot{\varphi }}_{\tau }\left(t\right)-f\left(\varphi_{\tau }\left(t\right)\right)\right\|}_{g(\varphi_{\tau }\left(t\right))}^{2}dt$$ (10)

where *f*(*φ*_*τ*_) is the deterministic field that describes the phenotypic landscape and from Eq (5) is,

$$\left(\begin{array}{c} f_{A}\left(x_{A},x_{B}\right)\\ f_{B}\left(x_{A},x_{B}\right) \end{array}\right)=\left(\begin{array}{c} p_{A}\left(M,x_{B}\right)-x_{A}\\ \alpha p_{B}\left(x_{A}\right)-\delta x_{B} \end{array}\right)$$ (11)

and the norm ${\left\|•\right\|}_{g(\varphi_{\tau })}^{2}$ in Eq (10) corresponds with the inner product 〈•,(*g*(*φ*_*τ*_)*g*(*φ*_*τ*_)^⊤^))^−1^•〉, where *g*(*φ*_*τ*_)*g*^⊤^(*φ*_*τ*_) ≡ *D* is the diffusion tensor given by the noise intensity that for Eq (5) reads,

$$D\left(x_{A},x_{B}\right)=\left(\begin{array}{cc} \nu_{A}p_{A}\left(M,x_{B}\right)+x_{A} & 0\\ 0 & \nu_{B}\alpha p_{B}\left(x_{A}\right)+\delta x_{B} \end{array}\right).$$ (12)

Thus, the value of the action can be obtained by the numerical minimisation Eq (9) of the action functional Eq (10) (see S1 Text). The result of this minimisation gives both the rate of stochastic switching for different values of the signal Eq (7) and the minimum action path (MAP) describing the transient expression profiles of *A* and *B* during the switching.

**Fig 5:**
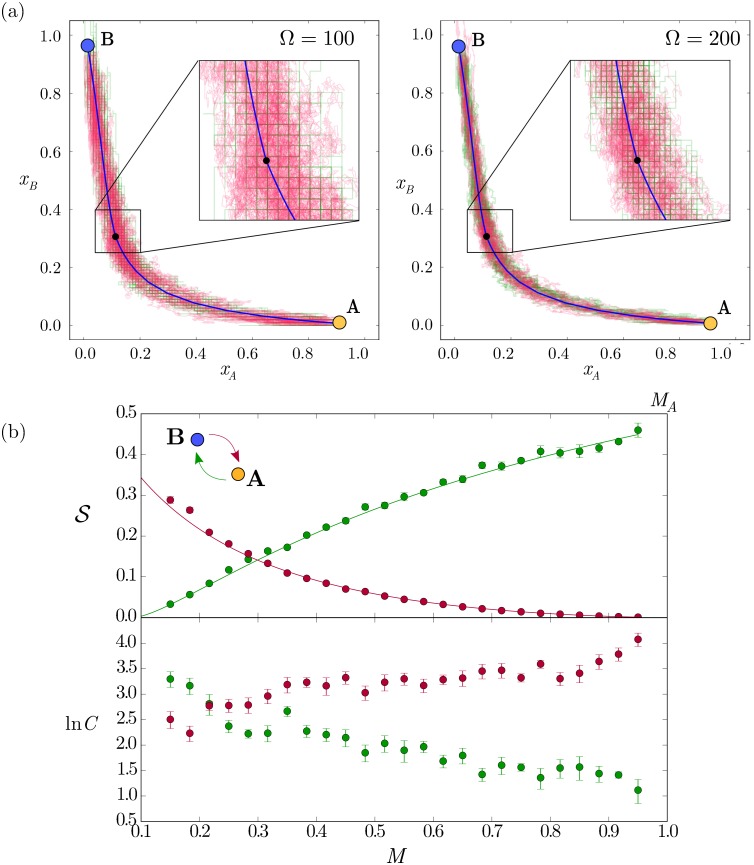
Stochastic switching trajectories concentrate along the MAP with rates that change along the bistable zone. (a) Stochastic switching trajectories are compared for CLE (red) and exact realisations of the kinetic reactions through the Gillespie algorithm (green) for two different values of Ω for the stochastic transition *A* → *B*. The trajectories concentrate along the MAP (solid blue line), which passes through the saddle point (black circle). This indicates that the MAP is consistent with the stochastic simulations. (b) The action S and the prefactor *C* depend on the level of the signal. Top) The value obtained for S by the minimisation of the action functional (solid lines) compared with the patterning times obtained for 10000 averaged switching CLE trajectories for each signal value (circles). Error bars indicate standard error from the fitting (see S1 Text). The logarithm of the switching times are lnC+ΩS, which predicts that switching rates from B to A increase dramatically with *M*, whilst the switching rate from A to B decrease, provided Ω is reasonably large. Parameters are those of Fig 4.

To test the validity of the action minimisation, we compared the MAP with the stochastic switching trajectories resulting from simulations of the kinetic reaction scheme (S1 Text) and the CLE Eq (5). This confirmed that stochastic trajectories concentrate around the MAP with increasing Ω (Fig 5(a)). This supports the validity of the MAP framework for estimating the trajectory of the transition and the computational efficiency, compared to stochastic simulations, makes it a useful complement to other techniques. Notably, the trajectory predicted by the MAP is distinct from the deterministic steepest-descent through the dynamical landscape given by the deterministic equations. The resulting path is shaped by the changes in intrinsic noise for different expression states through *g*(*φ*). Thus the MAP provides the means to explore the consequences of stochastic mechanisms, such as expression bursts, that are unavailable in deterministic descriptions.

In addition to the MAP, the validity of the action can be tested by comparing the switching times obtained from CLE stochastic realisations with the exponential dependence of switching time obtained from the action Eq (7) (Fig 5(b)). This shows that the action allows the patterning time to be determined with logarithmic precision for sufficiently large values of Ω, *i.e.* $\ln T_{s}=\ln C+\Omega S≃\Omega S$. Such values of Ω should involve patterning times greater than the patterning time in the monostable zone (Fig 5(b)). This reduces the necessity to determine the prefactor *C*, which is not given by the action minimisation. In cases where Ω is not large enough, the prefactor has a relevant contribution and can be obtained with the help of the minimised action, from a reduced number of CLE simulations (see S1 Text).

To characterise the effect of stochastic switching in the bistable zone, it is necessary to tally the change of fate *B* → *A* with its opposite *A* → *B* (Fig 5(b)). The shape of the MAP and the values of the action for transitions in both directions ($S_{AB}$ and $S_{BA}$) change along the bistable zone (Fig 5(b) and S4 Fig): transitions from A to B become less probable, and B to A more probable as *M* increases. This is translated as opposite trends of $S_{BA}$ and $S_{AB}$ with *M*. As a result, the residence time of the two states become equal ($S_{BA}≃S_{AB}$) at an intermediate value of the signal *M* ≃ 0.3. This predicts a new steady state position for the pattern boundary in the bistable zone. Away from this signal, the rate of one of the stochastic transitions becomes small compared with the other, resulting in one of the two states dominating. Strikingly, the location of the steady state boundary, whilst not dependent on Ω, is different to that predicted by the deterministic system at *M* = *M*_*A*_ = 1.0.

### Expression bursts shape the stochastic switching process but do not move the steady state boundary

One contributor to the stochastic nature of gene expression is the inherent pulsatility of transcription/translation—so-called ‘bursty’ expression—which results in sporadic interspersed periods of expression and quiescence [26, 30–32]. The framework we have developed allows us to explore the effect of the size of these bursts of expression, *ν*_*A*_ and *ν*_*B*_, on the behaviour of the system whilst keeping the deterministic dynamical landscape constant. The action is independent of Ω but is dependent on the burst sizes *ν*_*A*_ and *ν*_*B*_, which hence alter the MAP. The MAP approach is therefore suitable for capturing the differences in the effects of different noise sources.

**Fig 6:**
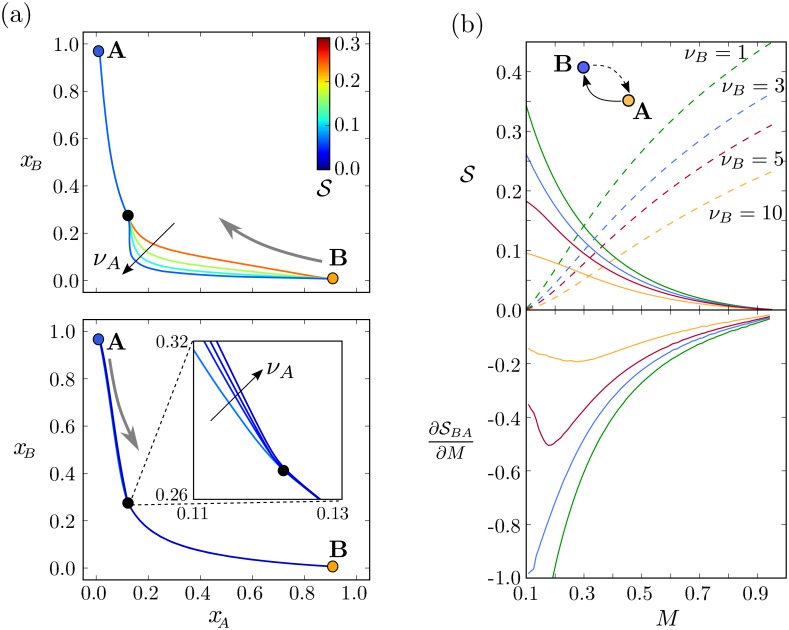
The stochastic switching trajectory and the rate change with the burst size. (a) Changing the relative burst size *ν*_*A*_ = 1, 3, 5, 10 for both switching processes *A* → *B* (top) and *B* → *A* (bottom) alters the MAP. The actions for the transitions (coloured lines) decrease and hence switching becomes more rapid as *ν*_*A*_ increases. The position of the different steady expression states are marked with colour circles for state A (orange) and B (blue) as well as the saddle points (black). Parameters are those of Fig 4. Morphogen signal is *M* = 0.45. For this value of *M* the effect on the action is more dramatic for the transition A→B. (b) Burst size modifies the change of action along the tissue. The action along the tissue evaluated for different values of *ν*_*B*_ (indicated by the different colours) for both switching transitions: *B* → *A* (solid line) and *A* → *B* (dashed lines) (top panel). The derivative of the action for *B* → *A* with respect to morphogen concentration level is shown in the bottom panel. Increasing the morphogen level biases the cells towards state A. The steady state position of the boundary is close to the value of *M* where dotted and dashed lines cross. The burst size has very little effect on these positions (value of *M*), but does affect how quickly they are approached. Parameters are those of Fig 4.

Intuitively, a larger burst size will introduce more noise in the expression of a protein and therefore facilitate a transition. This is confirmed quantitatively by comparing the actions of both switching processes (Fig 6(a) and S5 Fig), which reveals a reduction in the actions as *ν*_*A*_ and *ν*_*B*_ increase. In the particular case of Fig 6(a), the reduction in action for the transition A to B is much greater than the reduction in action for the reverse transition. This prompted us to investigate whether changes in burst size could modify the directionality of transitions.

Perturbations in the shape of the MAP are evident (Fig 6(a) and S5 Fig), suggesting that the greater the burst size of *A* the less activation of *B* is necessary for the repression of A. Equivalently, the greater the burst size of *B*, the less activation of A is necessary to repress B. This suggests that changes in *ν*_*A*_ and *ν*_*B*_ produce contrasting effects on the genetic profiles during switching. By contrast, a homogeneous increase in the noise, through a reduction in the typical number of molecules Ω, does not result in changes in the switching path.

A more detailed analysis of the effect of the burst sizes on the action profiles reveals that the approximate effect of an increase in burst size is to reduce both actions by a factor which is homogeneous across the tissue (Fig 6(b) and S6 Fig). This has the effect that, where $S_{BA}$ is larger than $S_{AB}$, the reduction in $S_{BA}$ as burst size increases is greater. By contrast, increases in burst size result in greater reduction in $S_{AB}$ where $S_{AB}$ is larger. Therefore, curiously, the burst parameters do not affect the directionality of the transitions, since they reduce the actions by the same amount at the steady state boundary position. At lower values of the morphogen signal, state B is still favoured, as at higher values is state A. In summary, different burst sizes produce different action profiles along the tissue (Fig 6(b)) and these will be translated into different transients and precisions of the boundary, but the steady state position of the boundary remains the same.

### The gene expression boundary propagates through the tissue at a velocity determined by stochastic effects

The action $S_{BA}$ grows superlinearly as the signal decreases (Fig 5(b)), and the stochastic switching time grows exponentially with Ω. This results in patterning times (residence time at *B* denoted by *T*_*B*_) that grow super-exponentially as the signal decreases. A consequence of this is that patterning time varies dramatically, differing by orders of magnitude, along the bistable zone. This can result in switching times much larger than those relevant to biological processes, in which case the steady state would never be reached. Such a big difference allows the separation of the bistable zone into two regions at any time *t* during the transient: an area where the stochastic switching time towards state A is much smaller than the current time, *T*_*B*_ ≪ *t*, (this region expresses predominantly *A*); and an area where the switching time towards state A is much larger than the current time, *T*_*B*_ ≫ *t*, (this region expresses predominantly *B*). The very large variation of *T*_*B*_ along the gradient *M* allows this separation, since the area of the tissue where *t* ≃ *T*_*B*_ is small. At different times the boundary between these two behaviours will be located at different spatial positions. Specifically, the boundary will be located at positions corresponding with values of the signal where switching time coincides with the current time *t* = *T*_*B*_, *i.e.* a value of the signal *M*_*s*_ where $S_{BA}=\frac{1}{\Omega }\ln\left(t/C\right)$. Thus, a bistable switch will produce a pattern boundary that advances away from the morphogen source with a velocity

$$\begin{array}{ccc} v(t) & = & \frac{dM_{s}}{dt}={\left({\left.\frac{dT_{B}}{dM}\right|}_{M_{s}}\right)}^{-1}=\\ & = & \frac{1}{t}{\left({\left.\frac{\partial \ln C}{\partial M}\right|}_{M_{s}}+\Omega {\left.\frac{\partial S_{BA}}{\partial M}\right|}_{M_{s}}\right)}^{-1}≃\frac{1}{\Omega t}{\left({\left.\frac{\partial S_{BA}}{\partial M}\right|}_{M_{s}}\right)}^{-1}, \end{array}$$ (13)

where, as described above, for large enough number of proteins (large Ω), the result is approximately independent of the prefactor *C*. The boundary velocity Eq (13) can therefore be determined by the value of Ω and by the dependence of the action on the signal, which is readily computed numerically (see Fig 6(b)). The greater the typical number of proteins, the slower the advance will be and in the deterministic limit (Ω → ∞), the boundary velocity vanishes.

**Fig 7:**
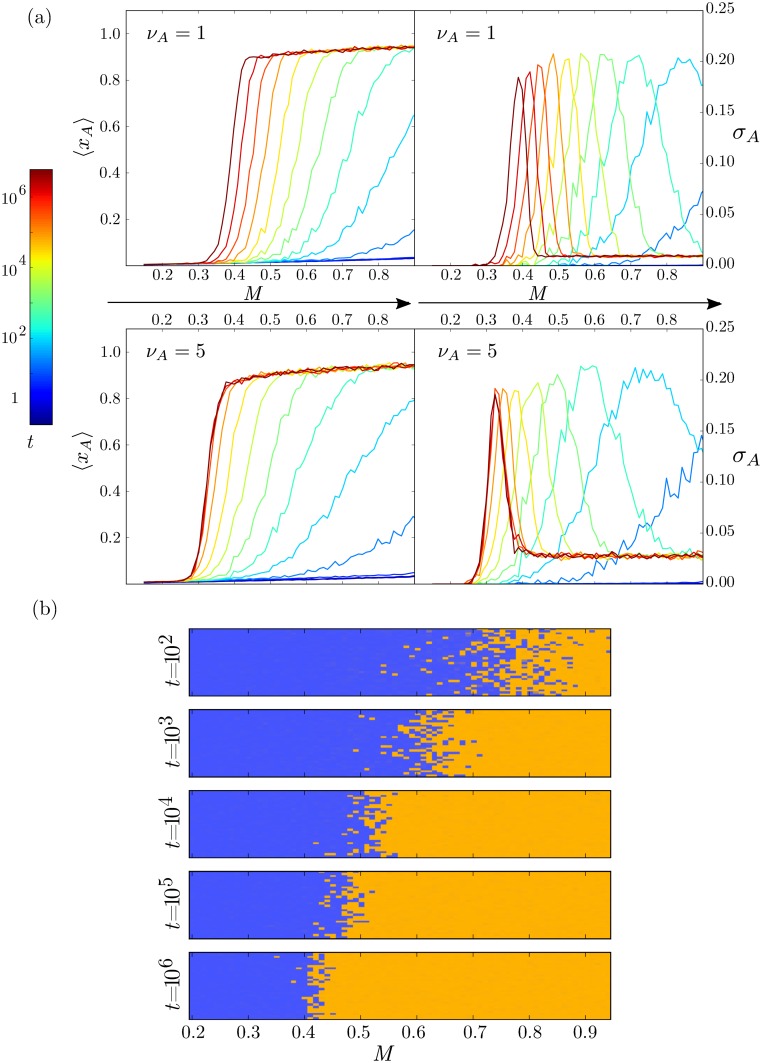
Switching events result in a travelling boundary with a speed depending on the stochastic parameters. (a) Mean and standard deviation of the expression of the morphogen activated gene *A* along the tissue at different time points for different burst sizes. Increasing the burst size makes the wave move faster but has a reduced impact on the precision of the boundary (see text for details). Results correspond to averaging of 500 trajectories with Ω = 120; the rest of the parameters are the same as in Fig 4. (b) Tissue simulations of the stochastic patterning process composed of 20 × 70 cells using trajectories from 7(a) top row (*ν*_*A*_ = 1). The colour of each cell is the RGB linear combination of blue and orange weighted with the expression states *x*_*A*_ and *x*_*B*_. Individual cells are observed to have a decisive state, expressing one of the transcription factors at a much higher level than the other. The boundary is not perfectly sharp but the region containing both cell types narrows over time.

The velocity of the advancing boundary Eq (13) decreases in time and the rate of this deceleration is given by the terms 1/*t* and $\frac{\partial S_{BA}}{\partial M}$, where the absolute value of this second term increases as the boundary propagates. As a result, the boundary can appear static for short periods of time, only revealing its movement when tracked for several orders of magnitude in time (Fig 7 and S1 Video). This is relevant for biological time scales, where a slowly travelling boundary may appear fixed. The position of the boundary at any time will be determined by Ω and the change of the action with the signal.

The travelling boundary induced by a graded morphogen signal can also be observed in stochastic simulations implemented on an array of cells (Fig 7 and S1 Video). Moreover, for each value of signal at each time point, the standard deviation in *x*_*A*_ between cells for each value of *M* can be measured. This gives an estimate of boundary precision. As expected the maximum variation is at the boundary *M*_*s*_ (Fig 7). Even at this position, however, the majority of individual cells are ‘decisive’, residing in either an A or B state and relatively few cells are in undefined/transition states where *x*_*A*_ ≃ *x*_*B*_ (Fig 7(b)). The decisiveness of individual cells is a consequence of the relatively rapid transition between states once initiated, compared with the stochastic switching time scale. Thus stochasticity results in a salt and pepper distribution of cell identities close to the boundary. The sharpness of the boundary during the transient at a time *t* is determined by the size of the zone where *t* ≃ *T*_*B*_, and will therefore increase its precision with Ω Eq (7). This indicates a trade-off between the sharpness of the boundary and the velocity towards the steady state (S7 Fig). The larger Ω, the more precise the boundary, but the slower the velocity of the boundary along the patterning axis (S8 Fig).

Finally, we asked how the burst size *ν*_*A*_ affects the dynamics of this travelling wave. Since alterations in *ν*_*A*_ produce different transition profiles $S_{BA}\left(M\right)$ (S6 Fig), this will affect the velocity of transitions. Specifically, an increase in burst size is predicted to increase the boundary velocity (Fig 7). As a result, even though different values of *ν*_*A*_ have the same steady state boundary *M* ≃ 0.3, they will have boundaries that travel along the patterning axis at different velocities. Strikingly, however, it appears (Fig 7), that the patterning precision, measured as the width of the region with elevated standard deviation in *x*_*A*_ between cells, is unaffected by the burst size, when comparing the patterns at a given time. This contrasts with the effect of Ω, where a smaller Ω always increases the width of the boundary at any time point in the transient (S8 Fig).

## Discussion

Our analysis suggests that gene expression noise profoundly affects key aspects of the dynamics and output of a morphogen controlled bistable toggle switch. A signature of deterministic models of a bistable switch is that at the threshold, *M*_*A*_, the system undergoes a saddle-node bifurcation. A consequence of this is that at values of *M* just greater than *M*_*A*_, the transient of the system is very slow [21]. Our analysis indicates that this slow dynamics of the deterministic bistable toggle switch, associated with the dynamical ghost, can be easily eliminated by the introduction of stochasticity into gene expression. In this situation, the fluctuations in gene expression allow a system close to *M* = *M*_*A*_ to escape spontaneously from this region of dynamical space, thereby speeding up cell identity determination in the monostable zone (*M* > *M*_*A*_).

Strikingly, stochastic and deterministic descriptions of the bistable switch also predict distinct final positions of the pattern. Intrinsic fluctuations result in stochastic switching events between states within the region of bistability (*M*_*B*_ < *M* < *M*_*A*_), which is not possible in the deterministic case. These rates change along the gradient with opposite trends and the steady state boundary for the stochastic system is located inside the bistable zone at the point where the rates of switching between the two states balance. This position, although different from the one defined by the deterministic system, does not change with increasing values of Ω. Moreover, this position is robust to expression burst size, and is therefore determined by the parameters describing the deterministic switch.

The duration of the transient towards the stochastic steady state is determined by the switching time. This varies super-exponentially along the tissue and results in a travelling front of switching in which the pattern boundary moves away from the morphogen source. The velocity of the advancing boundary can vary by orders of magnitude along the patterning axis, decreasing dramatically as the boundary propagates. This slowing of the movement means that the gene expression pattern in a tissue might never reach steady state within a biologically realistic time scale. In this scenario the tissue patterning is always effectively pre-steady state. As time progresses the slowing in boundary movement may mean that it appears effectively frozen, but in a different position from the steady state.

Hence, the model predicts the possibility of a biologically functional pattern different from steady state. The position and precision of the pattern at a particular time will be determined by the transition rates and these depend on the stochastic parameters comprising the typical number of proteins Ω and expression bursts size *ν*. Comparing the effects of altering these parameters suggests a trade off between distinct demands on the system. An increase in noise (smaller Ω) allows the system to approach steady state more rapidly. The consequence, however, is that the increased noise increases the rate of spontaneous switching and thereby decreases the precision of the gene expression boundary. By contrast, increase in burst size *ν* is predicted to accelerate pattern formation without the same deterioration in precision that results from decreasing the system size (Ω), at least at similar time points. Thus enhancing the bursting behaviour of gene expression might be one way to increase the speed at which the boundary advances through the tissue without the loss of precision resulting from decreasing the system size Ω. Alternatively, additional mechanisms might be employed to bypass any trade off. In situations in which a fast, precise pattern is required, differential cell adhesion, or other intercellular communication strategies, could be used to correct patterning mistakes [1, 14, 25]. In addition a morphogen signal that varies in time could be exploited, increasing the speed at which the steady state is approached. For example, a signal that reaches its peak amplitude rapidly after ligand stimulation and then progressively decreases might effectively increase the speed at which the final pattern is reached. In this respect, it is notable that two well studied morphogens, Tgf*β* and Shh, have both been reported to display the type of adapting signaling dynamics that could be well suited to this task [11, 52].

To explore the properties of the stochastic switching, the minimisation of the action proved an effective strategy. It provides a much more computationally efficient means to gather information about the stochastic properties of the system than conventional CLE or Gillespie integrations (see S1 Text). In cases in which the number of proteins is very small and the Eikonal assumption Eq (7) fails, further expansion in terms of powers of Ω can be used and large deviation principles still hold [33, 49]. The principal caveat of action minimisation strategies is the inability to find the prefactor *C* [16]. Nevertheless, even without the prefactor, the action allows time scales to be calculated with logarithmic precision and in many cases this is likely to be sufficient to identify the main patterning properties of biological tissues. For example we show that the dependence of the action on the level of morphogen allows the estimation of the velocity at which the pattern boundary moves.

The MAP approach also offers quantitative insight into the transient expression profiles of *A* and *B* during the stochastic switching between cell identities. The trajectory predicted by the MAP is distinct from that predicted by the steepest-descent of the deterministic landscape and is shaped by the burst size parameters (although not the system size parameter Ω). Stochastic simulations were consistent with the MAP, indicating that gene expression during individual cell switching can be predicted even when high levels of stochasticity are introduced. In this view, the dynamical landscape imposed by the regulatory interactions restricts the expression of cells as they transit between states to pass close to the saddle point in gene expression space. In addition, the transient path is similar in the mono- and bistable region of the system. This suggests that, in developing tissues, changes in cell identity should be characterised by defined trajectories in the levels of expression of the key genes. Thus even in the presence of gene expression noise, tightly constrained paths between the cell states would be representative of underlying kinetic mechanisms driving the cell state transition. This is consistent with ideas of developmental canalisation [55, 56] and leads to experimentally testable predictions that could be assessed using single cell resolution imaging of reporters for the two genes comprising the bistable switch. Observing coordinated changes in gene expression that matched MAP predictions would support the validity of the MAP approach and allow key parameters of the biological system to be estimated. Experimental corroboration of gene expression dynamics characteristic of those predicted by the MAP would provide insight into the mechanisms controlling cell decisions and greatly strengthen the evidence underpinning the use of dynamical systems theory to model these developmental events.

A practical feature of MAP theory is that it can be extended to larger networks comprising several genes and to full kinetic reaction schemes, that include, for example, the dynamics of mRNA production and decay and discrete promoter states as well as more detailed description of the expression bursts [16, 53, 54]. This would offer insight not only into simple binary decisions, such as that described in this study, but also provide a way to study transitions in more complex and realistic models of cell development [57, 58]. Thus MAP theory has the potential to provide a powerful framework to explore and understand the role of noise and dynamics in cell state transitions during normal development and also in other situations such as artificial directed reprogramming experiments [8, 58, 59]. More generally, our results emphasise that stochastic fluctuations in gene expression can influence the dynamics and outcome of gene regulatory networks and highlight the importance of developing the mathematical tools to explore these aspects of developmental patterning.

## Supporting Information

S1 Text Supplementary text. Technical details of the model including the detailed kinetic reactions of the bistable switch, the analysis of a switch without feedback, numerical integration of the stochastic trajectories and minimisation of the action.
(PDF)

S1 Video Stochastic patterning in the bistable zone. Trajectories correspond to the same simulations as in Fig 7. Time between frames increases exponentially.
(GIF)

S1 Fig Transient expression profiles for different values of morphogen signal *M*. a) Deterministic transient shows a slow down close to the threshold *M*_*A*_. b) Stochastic transient profiles to the steady state. Each line corresponds to one CLE realisation with a different signal input using same parameters from Fig 2 with *ν*_*A*_ = *ν*_*B*_ = 1 and Ω = 100.
(EPS)

S2 Fig Schematic representation of the cell fate dynamics for different values of the morphogen signal *M* around the threshold *M*_*A*_. For each morphogen value *M*, the velocity of change in genetic expression (green arrows) depends on the expression levels. (a) For low values of the signal in the bistable zone (*M*_*B*_ ≪ *M* ≪ *M*_*A*_), there are two well defined cellular states. (b) As the signal increases, the attraction towards the stable state B becomes weaker. (c) At the threshold *M*_*A*_ the stable minimum and the saddle collide cancelling each other (saddle-node bifurcation) resulting in a flat dynamical landscape for values *M* ≳ *M*_*A*_ with a very slow change in gene expression in time. (d) For higher morphogen signal the evolution towards the activated state A becomes faster.
(TIF)

S3 Fig Mean patterning time as a function of the system size Ω inside the bistable zone. Each point corresponds to the average mean first passage time of 100 CLE realisations. Inset) Coefficient of variation of the patterning times. Error bars correspond with standard error of the mean. Parameters are the same as in Fig 4.
(TIF)

S4 Fig Change of MAP along the bistable zone. MAPs correspond to 5 different values of the signal *M* for both transdifferentation processes *A* → *B* (top) and *B* → *A* (bottom). The value of the action (line colour) changes with the signal. The position of the different steady expression states are marked with colour circles for state A (orange) and B (blue) as well as the saddle points (black). The values of morphogen signal used are *M* = 0.10, 0.31, 0.52, 0.73, 0.95.
(TIF)

S5 Fig Stochastic switching trajectory and rate change with the burst size *ν*_*B*_. Change of MAP for different values of the relative burst size *ν*_*B*_ = 1, 3, 5, 10 for both switching processes *A* → *B* (top) and *B* → *A* (bottom). The value of the action (line colour) changes with *ν*_*B*_. The position of the different steady expression states are marked with colour circles for state A (orange) and B (blue) as well as the saddle points (black). Morphogen signal is *M* = 0.45. Parameters are those of Fig 4.
(TIF)

S6 Fig Burst size *ν*_*A*_ modifies the change of action along the tissue. The action along the tissue is evaluated for different values of *ν*_*A*_ for both switching transitions: *B* → *A* (solid line) and *A* → *B* (dashed line), and its derivative over the morphogen concentration (bottom panel), vary as a function of the morphogen, revealing the time scale differences and directionality during the patterning process. Parameters are those of Fig 4.
(TIF)

S7 Fig Position of the boundary towards the steady state depends on the typical number of proteins. Position of the boundary is measured as the value of the signal for which 〈*x*_*A*_〉 = 0.5 and error bars indicate the ranges 〈*x*_*A*_〉 = [0.4, 0.6]. Each point is the average of 200 stochastic trajectories. Parameters are the same as in Fig 4.
(TIF)

S8 Fig Travelling boundary velocity and precision depend on system size Ω. Mean and standard deviation in expression of the morphogen activated gene *A* along the tissue at different time points for different values of Ω. Results correspond to averaging of 500 trajectories with *ν*_*A*_ = *ν*_*B*_ = 1; the rest of the parameters are the same as in Fig 4.
(TIF)
