# Correction: Intrinsic Noise Profoundly Alters the Dynamics and Steady State of Morphogen-Controlled Bistable Genetic Switches

**DOI:** 10.1371/journal.pcbi.1005563

**Published:** 2017-05-26

**Authors:** The PLOS Computational Biology Staff

The funding statement for this article should read as follows:

“This work was supported by the Francis Crick Institute which receives its core funding from Cancer Research UK (FC001051), the UK Medical Research Council (FC001051), and the Wellcome Trust (FC001051); also supported by the Wellcome Trust (grant references WT098325MA and WT098326MA). The funders had no role in study design, data collection and analysis, decision to publish, or preparation of the manuscript.”

## References

[1] Perez-Carrasco R, Guerrero P, Briscoe J, Page KM (2016) Intrinsic Noise Profoundly Alters the Dynamics and Steady State of Morphogen-Controlled Bistable Genetic Switches. PLoS Comput Biol 12(10): e1005154 doi:10.1371/journal.pcbi.1005154 27768683 10.1371/journal.pcbi.1005154 PMC5074595

