# Supplementary material for: Intrinsic Noise Profoundly Alters the Dynamics and Steady State of Morphogen-Controlled Bistable Genetic Switches
Source: PLoS Comput Biol. 2016 Oct 21;12(10):e1005154. doi: 10.1371/journal.pcbi.1005154 (PMC5074595; doi:10.1371/journal.pcbi.1005154)
Supplement: S1 Text — Technical details of the model including the detailed kinetic reactions of the bistable switch, the analysis of a switch without feedback, numerical integration of the stochastic trajectories and minimisation of the action. (PDF) [file pcbi.1005154.s001.pdf]

Intrinsic noise profoundly alters the dynamics and steady state of morphogen-controlled bistable genetic switches

Supplementary Text S1:

Kinetic reaction scheme of the bistable switch

The reaction kinetics of the bistable switch consist of the production and degradation reaction for each gene A, B as,

$$\varnothing \xrightarrow{\tilde{\Omega}\alpha_A p_A/\nu_A} \nu_A A, \qquad A \xrightarrow{\delta_A} \varnothing$$
$$\varnothing \xrightarrow{\tilde{\Omega}\alpha_B p_B/\nu_B} \nu_B B, \qquad B \xrightarrow{\delta_B} \varnothing. \quad \text{(ST.1)}$$

where the bursty expression of each gene is introduced in a first approximation as a stoichiometry in the production reactions. More complete descriptions of the kinetic reactions would involve mRNA dynamics, promoter state dynamics or protein-protein interactions. The Chemical Langevin Equation (5) is the limiting description of this reaction scheme [1]. The CLE approximation will break down if the reaction rates change significantly within the time scale of the slowest reaction, and hence full simulation of the reaction kinetics (ST.1) is required [1]. Comparison between full simulations of (ST.1) and the solution of the CLE are presented in Fig. 5 showing the validity of the approximation for the biological scenario studied.

Additionally, it is also interesting to point out that the regulatory functions described in (4) are in fact the binding probabilities for a haploid system. As long as the number of molecules of the relevant transcription factors and polymerase are not very low (assumptions already necessary to the derivation of (4)), in diploid cells, the kinetic equations are the same, subject to a doubling of the values of α_i. The interpretation of the terms in (4) is slightly different since the production rate is proportional to $2 \cdot \text{Prob(both alleles of promoter bound)}+$ Prob(one allele of promoter bound). This statement also extends to the stochastic formulation where the same CLE is obtained.

Patterning without positive feedback loop $M \to A$

To illustrate the effect on patterning time imposed by the regulatory architecture of the bistable switch, we compared it with a regulatory motif which lacks the cross repressive feedback between A and B and instead relies on strongly cooperative activation of A by M. In this non-feedback case, there is only one steady state and the expression level of A has a sigmoidal dependence on the level of morphogen, so A appears absent for low morphogen values, while A is expressed for high morphogen values.

In order to get a step-like sigmoidal response to the signal for a monostable system, it is necessary to use high cooperativity for the morphogen activation. In the context of the promoter state using the thermodynamical description this can be introduced as h independent binding sites for the activating signal on the promoter each one facilitating the binding of the RNAp when bound by the signal [2],

$$p_A^*(M) = \left(1+\rho_A\left(1+\frac{x_B}{K_B}\right)^2\left(\frac{1+M/K_M}{1+fM/K_M}\right)^h\right)^{-1}. \quad \text{(ST.2)}$$

Even though the feedback loop is removed, a certain constant amount of x_B is considered to represent the boundary position at the same signal value. Alternatively this is equivalent to redefining the value of ρ_A. For the comparison in the text, the values used are $h = 25$, $x_B = 3$ and $K_B = 0.12$ which give a sharp boundary that reaches its maximum expression at $M \simeq 1$ (Fig. ST.1). The rest of the parameters are the same as the complete bistable switch to facilitate their comparison (see Fig. 2).

We defined this model to generate an outcome that resembles the bistable switch case although quantitative comparison with the feedback case is limited by the specific parameters chosen. Nevertheless, the results suggest that, in the absence of feedback, patterning time is in general faster than the bistable switch for all values of the signal. Importantly, since the stable state never loses stability there is no dramatic increase in patterning time close to the boundary M_A (Fig. ST.2). As a consequence, considering the intrinsic fluctuations of the system does not introduce any dramatic change in the patterning time (Fig. ST.3).

More precisely, the deterministic non-feedback circuit dynamics are determined by the linear ODE,

$$\dot{n}_A = \alpha_A p_A^* - \delta_A n_A \quad \text{(ST.3)}$$

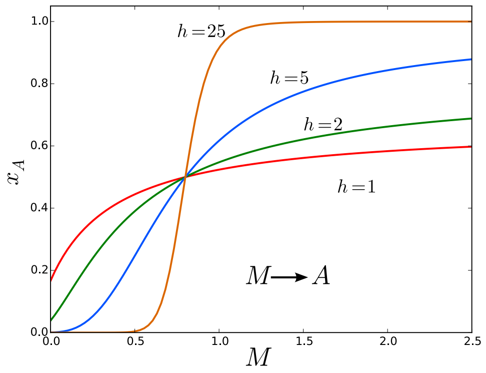

Figure ST.1. Stationary expression of A without the positive feedback loop for different values of the cooperativity exponent h.

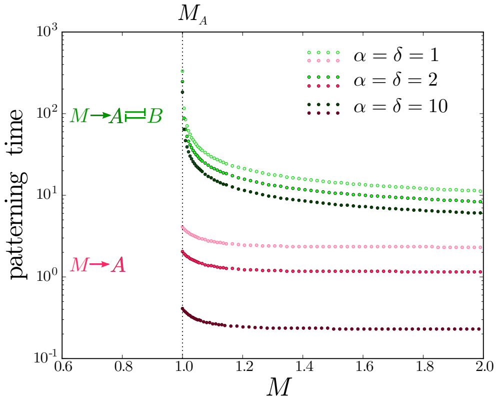

Figure ST.2. Variation of deterministic patterning time along the tissue is intrinsic to the bistable switch. The bistable switch (green circles) is compared with the non-feedback case (magenta circles) for three different values of $\alpha_B = \delta_B = \{1, 2, 10\}$. For signal values below the threshold signal M_A (dotted line), state A is never reached. The patterning time is measured as the time necessary to observe a response $x_A > 0.9$. Since the parameters which vary in this figure are α_B and δ_B, the nondimensionalisation is not affected. Therefore changes in nondimesional times correspond simply with changes in dimensional times. Other parameters are the same as in Fig. 2.

This predicts an exponential decay that for the initial condition $n_A = 0$ is,

$$n_A(M,t) = \frac{\alpha_A p_A^*}{\delta_A}\left(1 - \mathrm{e}^{-\delta_A t}\right). \qquad \text{(ST.4)}$$

The comparison in dynamical response of the bistable switch to the non-feedback motif can be extended to changes in parameters α and δ, the relative production and degradation rates respectively. Concretely, keeping the ratio α/δ fixed, the response speed of gene A over B can be controlled. In both cases, reducing the time scale response of B speeds up the whole transient and reduces the patterning time (Fig. ST.2). In the case of the non-feedback model the reduction of patterning time is linear with respect to changes in α and δ. By contrast, the patterning time of the bistable switch is much more resilient to changes in α and δ (Fig. ST.2). This feature provides some robustness in patterning time to changes in parameters and means that the bistable switch is intrinsically slower than the non-feedback mechanism.

Numeric minimization of the Action

The minimisation of the action (9) was performed by discretising the continuous path φ_τ in N straight segments between positions $\{\phi_0, \phi_1, \phi_2, \ldots\phi_N\}$ of duration Δt. With this approximation, the action (10) can be written as,

$$\mathcal{S}(\varphi_\tau) = \frac{1}{2}\sum_{k=1}^{N}\sum_{i=\{A,B\}}\frac{\left(\dot\varphi_k^i - f_k^i\right)^2}{D_k^i}\Delta t, \qquad \text{(ST.5)}$$

where the index i runs over the two genes and $k = 1, \ldots, N$ over each linear segment of the path. D_k^i and f_k^i are the ith component of the diagonal diffusion tensor (12) and the deterministic field (11) for each segment evaluated at its centre $x_k = (\phi_{k-1} + \phi_k)/2$. Additionally, the velocity along a segment is $\dot\varphi_k = (\phi_k - \phi_{k-1})/\Delta t$. For a certain fixed total time τ and fixed initial and final position φ_0 and φ_N, the discretised action (ST.5) for a path can be minimised using a quasi-Newtonian method with the help of the analytical expression for the derivative of the action on the direction of the l-th

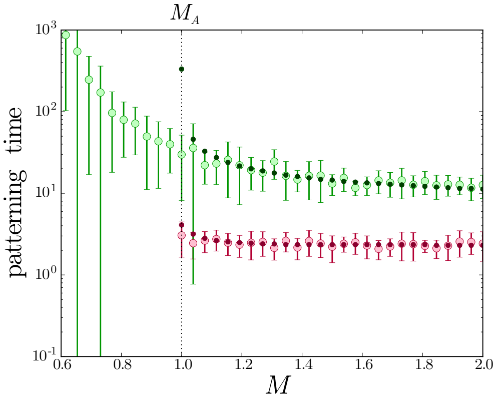

Figure ST.3. Patterning circuit without feedback is not affected by intrinsic noise. Patterning time for different levels of the signal for a case with intrinsic noise (big circles) and the deterministic case (small circles). The bistable switch (green circles) is compared with the non-feedback case (magenta circles). The patterning time is measured as the time necessary to observe a stationary response $x_A > 0.9$. There is no patterning transient for the case without feedback for signal values below the M_A (dotted line) since the initial state (phenotype B) is already the steady state phenotype. Each point corresponds to the average of 15 CLE realisations with standard deviation marked with error bars. Parameters are the same as in Fig. 4.

genetic component of segment point s,

$$\begin{aligned} \frac{\partial \mathcal{S}}{\partial \phi_s^l} &= \frac{\dot{\varphi}_s^l - f_s^l}{D_s^l} - \frac{\dot{\varphi}_{s+1}^l - f_{s+1}^l}{D_{s+1}^l} \\ &- \frac{\Delta t}{2} \sum_{j=\{A,B\}} \left[\frac{\dot{\varphi}_{s+1}^j - f_{s+1}^j}{D_{s+1}^j} f'^j_{l,s+1} \right. \\ &+ \frac{\dot{\varphi}_s^j - f_s^j}{D_s^j} f'^j_{l,s} \\ &+ \frac{(D')^j_{l,s+1}}{2(D^2)^j_{l,s+1}} \left(\dot{\varphi}_{s+1}^j - f_{s+1}^j \right)^2 \\ &+ \left. \frac{(D')^j_{l,s}}{2(D^2)^j_{l,s}} \left(\dot{\varphi}_s^j - f_s^j \right)^2 \right] \end{aligned} \quad \text{(ST.6)}$$

where $D'^j_{l,s}$ and $f'^j_{l,s}$ are the derivative in the l direction of the jth component of the diffusion tensor (12) and the deterministic field (11) of segment s. The minimisation of the action with $2 \times (N-1)$ degrees of freedom was performed using the LFBGS minimisation strategy. The resulting value of the action S_τ is the minimum action for a certain path time τ. The optimal action is found by minimising the resulting S_τ with τ by means of a simple custom made line search method. To speed up the convergence to the solution the starting path consists of two straight lines joining the initial and final phenotype with the saddle point. The number of segments for the simulations used in the optimisation is $N = 150$, a larger number of segments did not exhibit an improvement in the precision of the action while increasing dramatically the computational time.

The actions for both switching directions $\mathcal{S}_{AB}$ and $\mathcal{S}_{BA}$ is determined by the boundary conditions of the integration fixing ϕ_0 and ϕ_N at the initial and final expression amounts of the corresponding bistable states A and B. In order to obtain the expression value of the bistable states for different morphogen signals, the bistable switch was reconstructed using continuation strategies through the *PyDSTool* environment [3].

Simulation of stochastic trajectories

Simulations of the stochastic trajectories were performed using custom made code. Realisations of the Chemical Langevin Equation have been integrated using the Euler-Maruyama algorithm that reproduces the discrete Wiener process [4,5], while the exact realisations of the kinetic reaction equations were performed using the Gillespie algorithm [6]. Both methods require the reproduction of the whole trajectory and the computing time will be proportional to the trajectory time making these calculations very slow when computing stochastic switching events with increasing number of proteins Ω. By contrast, the computation of the action is immediate, independent of the actual transition time between states, making it a useful tool for studying spontaneous cell fate change.

The main drawback in computing the action is the unknown prefactor C (7). This means that the transition time can be computed only to logarithmic precision [7,8]. In cases in which a greater precision is required, the prefactor C can be obtained by fitting an exponential to average transition times obtained with the CLE or Gillespie at adequate values of Ω, such that Ω is large enough that the behaviour can be considered exponential, and low

enough to obtain reasonable computation times. This range can be guessed from the values of the action $\mathcal{S}$ while checking the goodness of the exponential fit. For the case of Fig. 5 the prefactor C was computed by choosing a range where the mean first passage time depended exponentially on Ω, and performing a least squares fitting to an exponential function $T = C\mathrm{e}^{\Omega\mathcal{S}}$. The least squares fitting was not linearized to give more weight to points of large Ω, where the exponential behaviour is assured.

References

1. Gillespie DT. The chemical Langevin equation. The Journal of Chemical Physics. 2000;113(1):297.

2. Bintu L, Buchler NE, Garcia HG, Gerland U, Hwa T, Kondev J, et al. Transcriptional regulation by the numbers: Models. Current Opinion in Genetics and Development. 2005;15(2):116–124.

3. Clewley R. Hybrid Models and Biological Model Reduction with PyDSTool. PLoS Computational Biology. 2012 Aug;8(8):e1002628.

4. Maruyama G. Continuous Markov processes and stochastic equations. Rend Circ Mat Palermo. 1955;10:48.

5. Garcia-Ojalvo J, Sancho J. Noise in Spatially Extended Systems. Institute for Nonlinear Science. Springer New York; 2012.

6. Gillespie DT. Exact stochastic simulation of coupled chemical reactions. The Journal of Physical Chemistry. 1977 Dec;81(25):2340–2361.

7. Touchette H. The large deviation approach to statistical mechanics. Physics Reports. 2009;478(1-3):1–69.

8. Lv C, Li X, Li F, Li T. Constructing the Energy Landscape for Genetic Switching System Driven by Intrinsic Noise. PLoS ONE. 2014;9(2):e88167.
