# Supplementary figures and images for: Intrinsic Noise Profoundly Alters the Dynamics and Steady State of Morphogen-Controlled Bistable Genetic Switches
Source: PLoS Comput Biol. 2016 Oct 21;12(10):e1005154. doi: 10.1371/journal.pcbi.1005154 (PMC5074595; doi:10.1371/journal.pcbi.1005154)

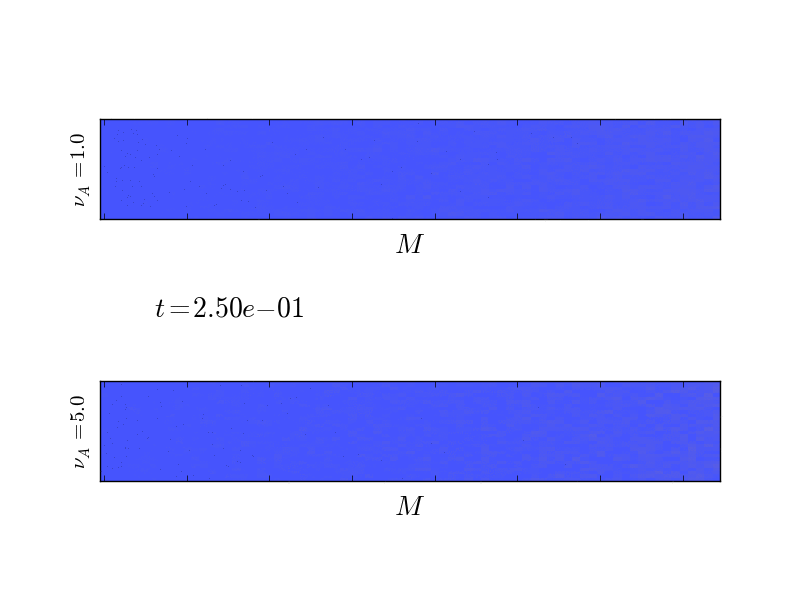

Supplement: S1 Video — Trajectories correspond to the same simulations as in Fig 7. Time between frames increases exponentially. (GIF) [file pcbi.1005154.s002.gif]

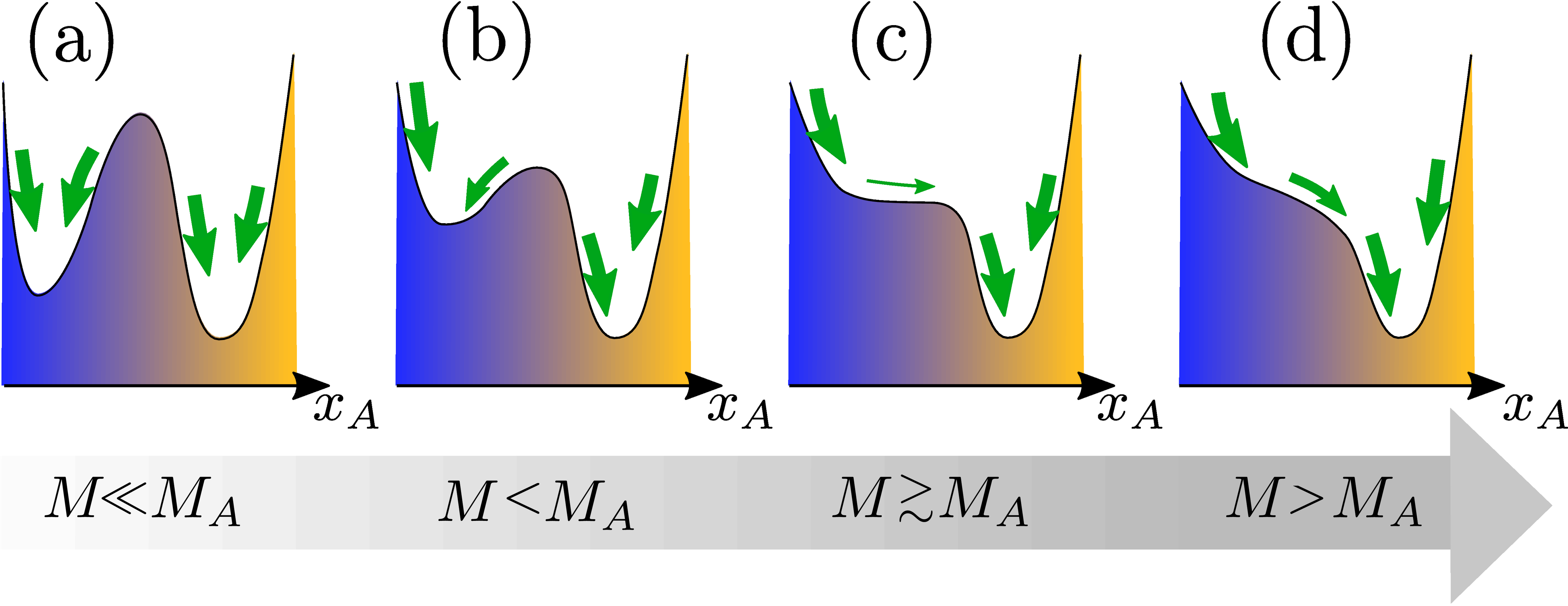

Supplement: S2 Fig — For each morphogen value M, the velocity of change in genetic expression (green arrows) depends on the expression levels. (a) For low values of the signal in the bistable zone (MB ≪ M ≪ MA), there are two well defined cellular states. (b) As the signal increases, the attraction towards the stable state B becomes weaker. (c) At the threshold MA the stable minimum and the saddle collide cancelling each other (saddle-node bifurcation) resulting in a flat dynamical landscape for values M ≳ MA with a very slow change in gene expression in time. (d) For higher morphogen signal the evolution towards the activated state A becomes faster. (TIF) [file pcbi.1005154.s004.tif]

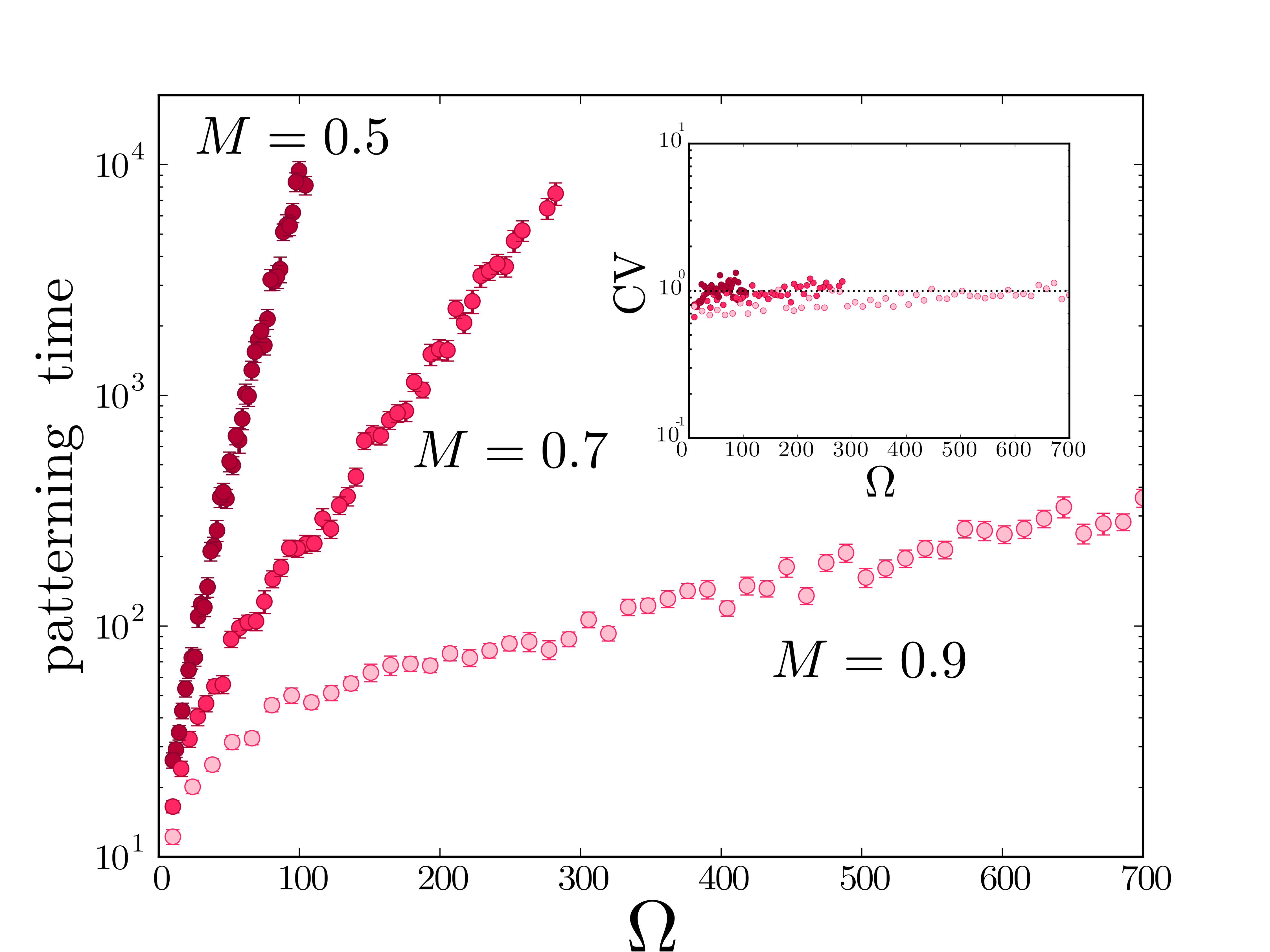

Supplement: S3 Fig — Each point corresponds to the average mean first passage time of 100 CLE realisations. Inset) Coefficient of variation of the patterning times. Error bars correspond with standard error of the mean. Parameters are the same as in Fig 4. (TIF) [file pcbi.1005154.s005.tif]

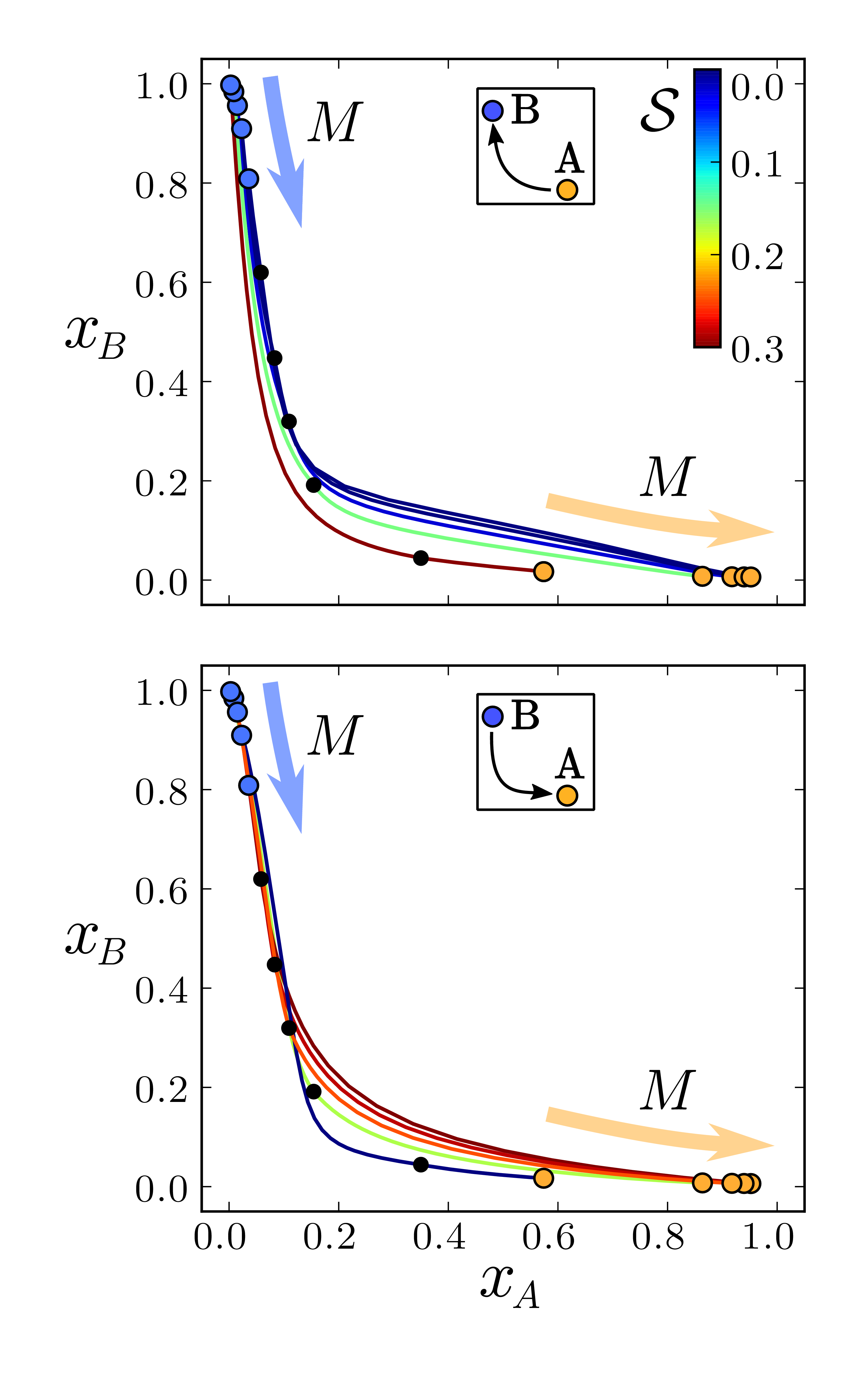

Supplement: S4 Fig — MAPs correspond to 5 different values of the signal M for both transdifferentation processes A → B (top) and B → A (bottom). The value of the action (line colour) changes with the signal. The position of the different steady expression states are marked with colour circles for state A (orange) and B (blue) as well as the saddle points (black). The values of morphogen signal used are M = 0.10, 0.31, 0.52, 0.73, 0.95. (TIF) [file pcbi.1005154.s006.tif]

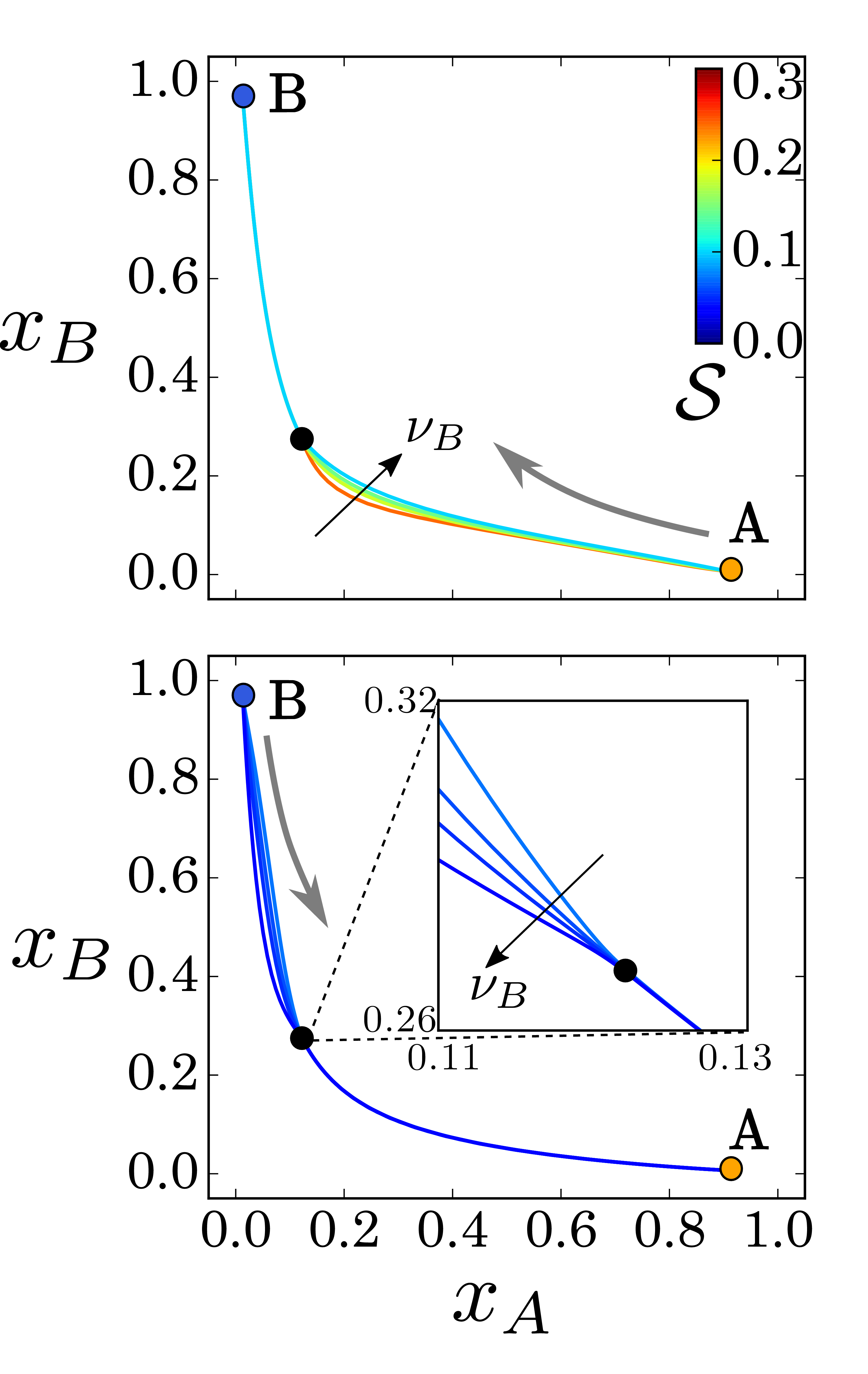

Supplement: S5 Fig — Change of MAP for different values of the relative burst size νB = 1, 3, 5, 10 for both switching processes A → B (top) and B → A (bottom). The value of the action (line colour) changes with νB. The position of the different steady expression states are marked with colour circles for state A (orange) and B (blue) as well as the saddle points (black). Morphogen signal is M = 0.45. Parameters are those of Fig 4. (TIF) [file pcbi.1005154.s007.tif]

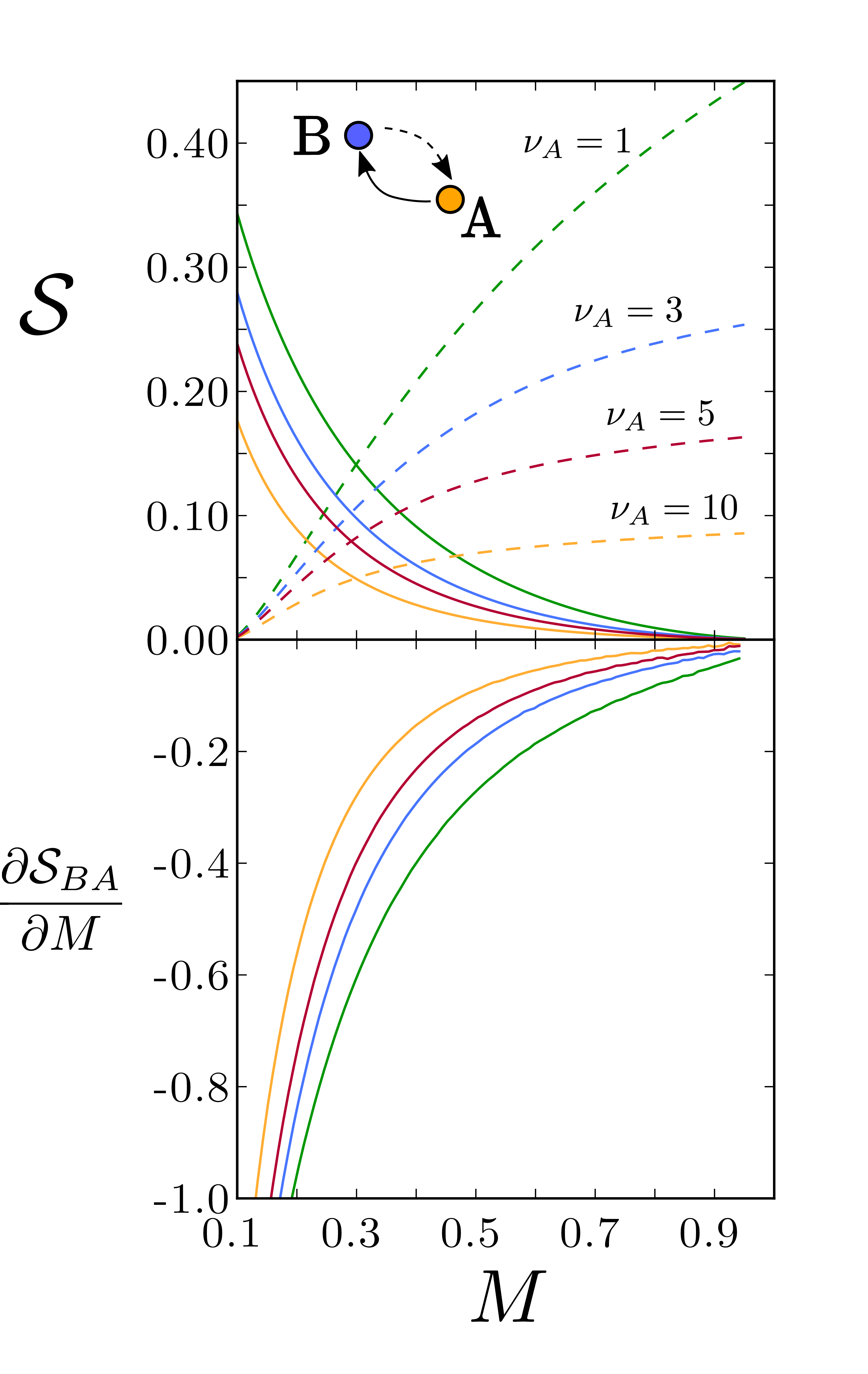

Supplement: S6 Fig — The action along the tissue is evaluated for different values of νA for both switching transitions: B → A (solid line) and A → B (dashed line), and its derivative over the morphogen concentration (bottom panel), vary as a function of the morphogen, revealing the time scale differences and directionality during the patterning process. Parameters are those of Fig 4. (TIF) [file pcbi.1005154.s008.tif]

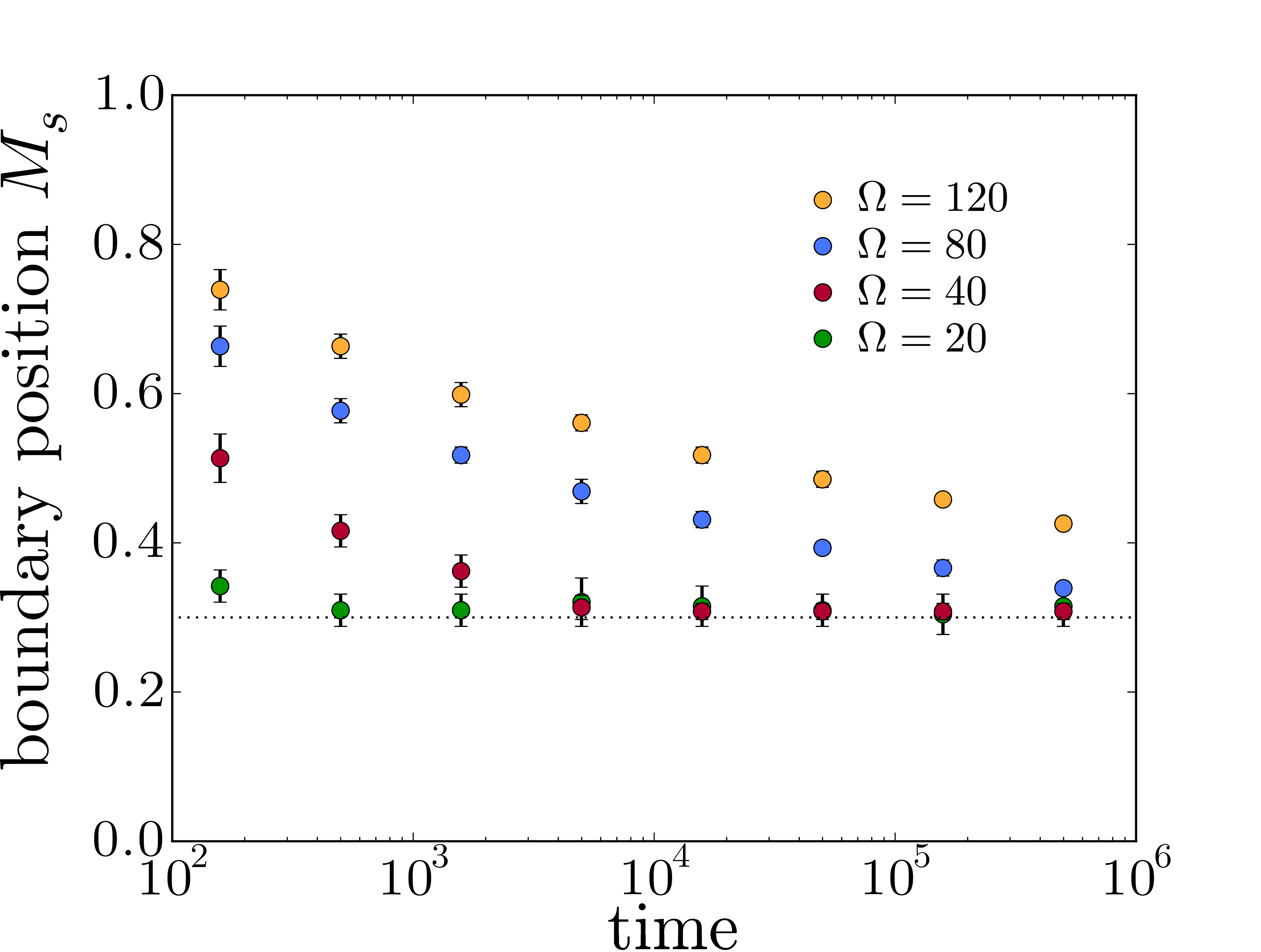

Supplement: S7 Fig — Position of the boundary is measured as the value of the signal for which 〈xA〉 = 0.5 and error bars indicate the ranges 〈xA〉 = [0.4, 0.6]. Each point is the average of 200 stochastic trajectories. Parameters are the same as in Fig 4. (TIF) [file pcbi.1005154.s009.tif]

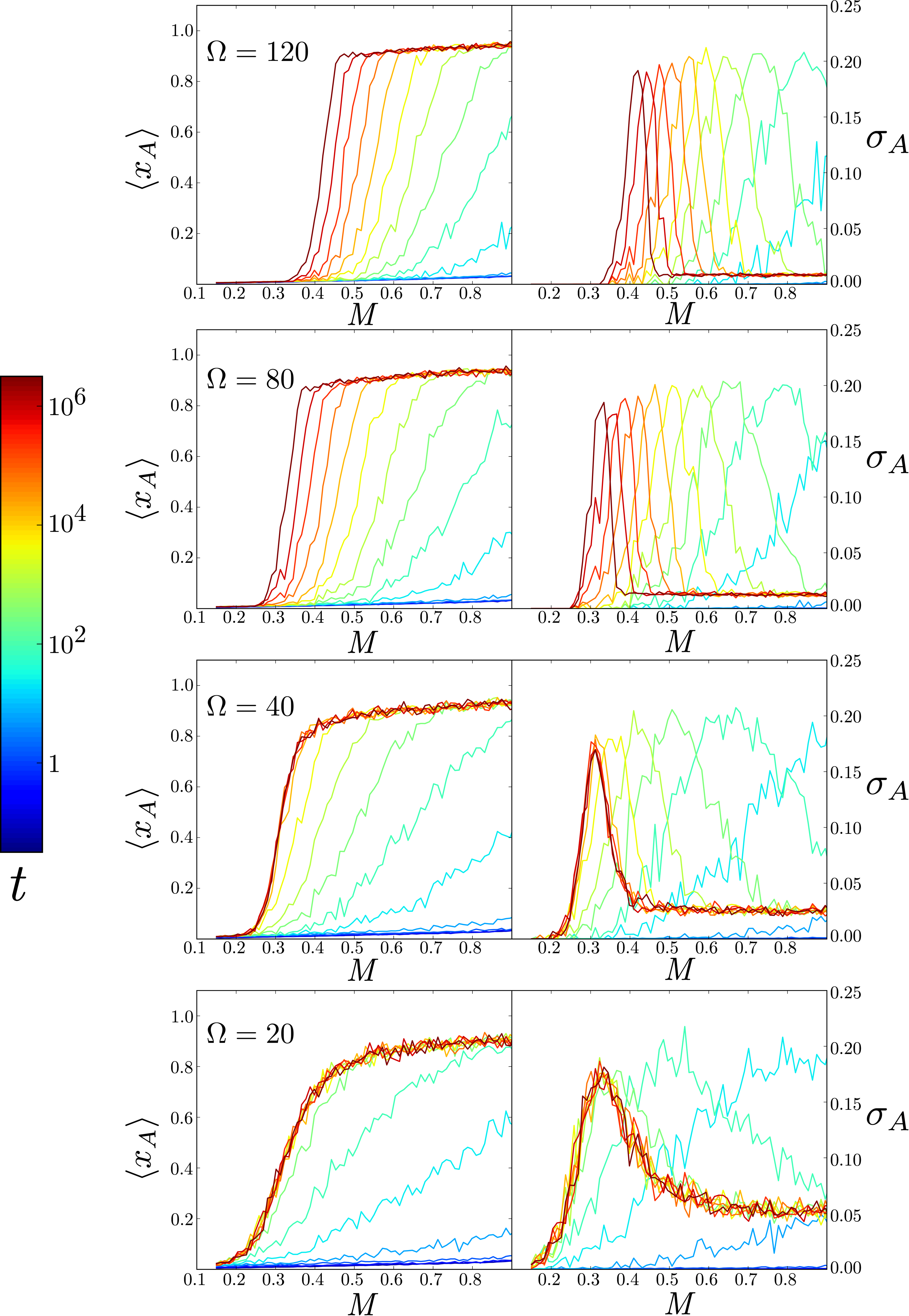

Supplement: S8 Fig — Mean and standard deviation in expression of the morphogen activated gene A along the tissue at different time points for different values of Ω. Results correspond to averaging of 500 trajectories with νA = νB = 1; the rest of the parameters are the same as in Fig 4. (TIF) [file pcbi.1005154.s010.tif]
